# Connectivity and systemic resilience of the Great Barrier Reef

**DOI:** 10.1371/journal.pbio.2003355

**Published:** 2017-11-28

**Authors:** Karlo Hock, Nicholas H. Wolff, Juan C. Ortiz, Scott A. Condie, Kenneth R. N. Anthony, Paul G. Blackwell, Peter J. Mumby

**Affiliations:** 1 Marine Spatial Ecology Lab, School of Biological Sciences, The University of Queensland, Brisbane St Lucia, Australia; 2 CSIRO Oceans & Atmosphere, Hobart, Australia; 3 Australian Institute of Marine Science, Townsville, Australia; 4 School of Mathematics & Statistics, University of Sheffield, Sheffield, United Kingdom; 5 ARC Centre of Excellence for Coral Reef Studies, The University of Queensland, Brisbane St Lucia, Australia; Simon Fraser University, Canada

## Abstract

Australia’s iconic Great Barrier Reef (GBR) continues to suffer from repeated impacts of cyclones, coral bleaching, and outbreaks of the coral-eating crown-of-thorns starfish (COTS), losing much of its coral cover in the process. This raises the question of the ecosystem’s systemic resilience and its ability to rebound after large-scale population loss. Here, we reveal that around 100 reefs of the GBR, or around 3%, have the ideal properties to facilitate recovery of disturbed areas, thereby imparting a level of systemic resilience and aiding its continued recovery. These reefs (1) are highly connected by ocean currents to the wider reef network, (2) have a relatively low risk of exposure to disturbances so that they are likely to provide replenishment when other reefs are depleted, and (3) have an ability to promote recovery of desirable species but are unlikely to either experience or spread COTS outbreaks. The great replenishment potential of these ‘robust source reefs’, which may supply 47% of the ecosystem in a single dispersal event, emerges from the interaction between oceanographic conditions and geographic location, a process that is likely to be repeated in other reef systems. Such natural resilience of reef systems will become increasingly important as the frequency of disturbances accelerates under climate change.

## Author summary

Australia’s Great Barrier Reef is a large coral ecosystem consisting of more than 3,800 reefs. Coral populations inhabiting these reefs are connected by larvae that are dispersed by ocean currents. Modelling regional connectivity patterns reveals reefs that can act as prominent larval sources and supply larvae to other coral populations in the area. Coral populations on reefs are also subject to various disturbances, such as bleaching and outbreaks of the coral-eating crown-of-thorns starfish. These disturbances tend to have spatially explicit patterns, resulting in different levels of impact among reefs. In this study, we first use high-resolution dispersal simulations of larvae to identify the reefs most likely to support regional recovery processes due to their high connectivity. We then use oceanographic and climate models to show which reefs are likely to have a lower risk of exposure to coral bleaching and starfish outbreaks. Finally, we combine these results to find reefs that are not only likely to be good sources by being well connected but also more likely to have adult breeding stocks needed to provide the necessary larval supply. This information can support decision-making that aims to allocate management resources and prioritise sites important for the resilience of the entire reef system.

## Introduction

Marine ecosystems are characterised by high levels of larval connectivity among populations linked by ocean or coastal currents [1]. Ensuring functioning and resilient ecosystems requires that processes of connectivity are maintained, particularly when the metapopulation has been widely depleted and individual patches must recolonise from neighbours [2]. Circumstances of large-scale metapopulation depletions are commonly found on coral reefs that are vulnerable to mass thermal stress events that elicit coral bleaching on scales of hundreds to thousands of kilometres [3–5]. Yet much of the science of coral reef resilience has focused on reducing the exposure of reefs to stressful conditions, be they physical pollutants or the impacts of ecosystem exploitation [6–8]. While the importance of regional connectivity of larvae is a widely recognised process of recovery [9,10], it has rarely been operationalised for building resilient ecosystems, although methods exist [11,12]. In principle, marine reserve networks might incorporate the most important sources of replenishment with a view to promoting region-wide recovery after disturbance [12–16]. We refer to this process as building systemic resilience, in the sense that protecting these sources will promote resilience of a wider system and facilitate metapopulation recovery after major disturbances [17]. Much of the discussion of systemic resilience has focused on recognizing the cascades of failures that could lead to catastrophic transitions of the entire system to an undesirable state [17]. Here, however, we focus on the recovery side of the story, looking for potential local refugia that can drive cascading processes of large-scale recovery and provide novel intervention points for ecosystem management.

**Fig 1:**
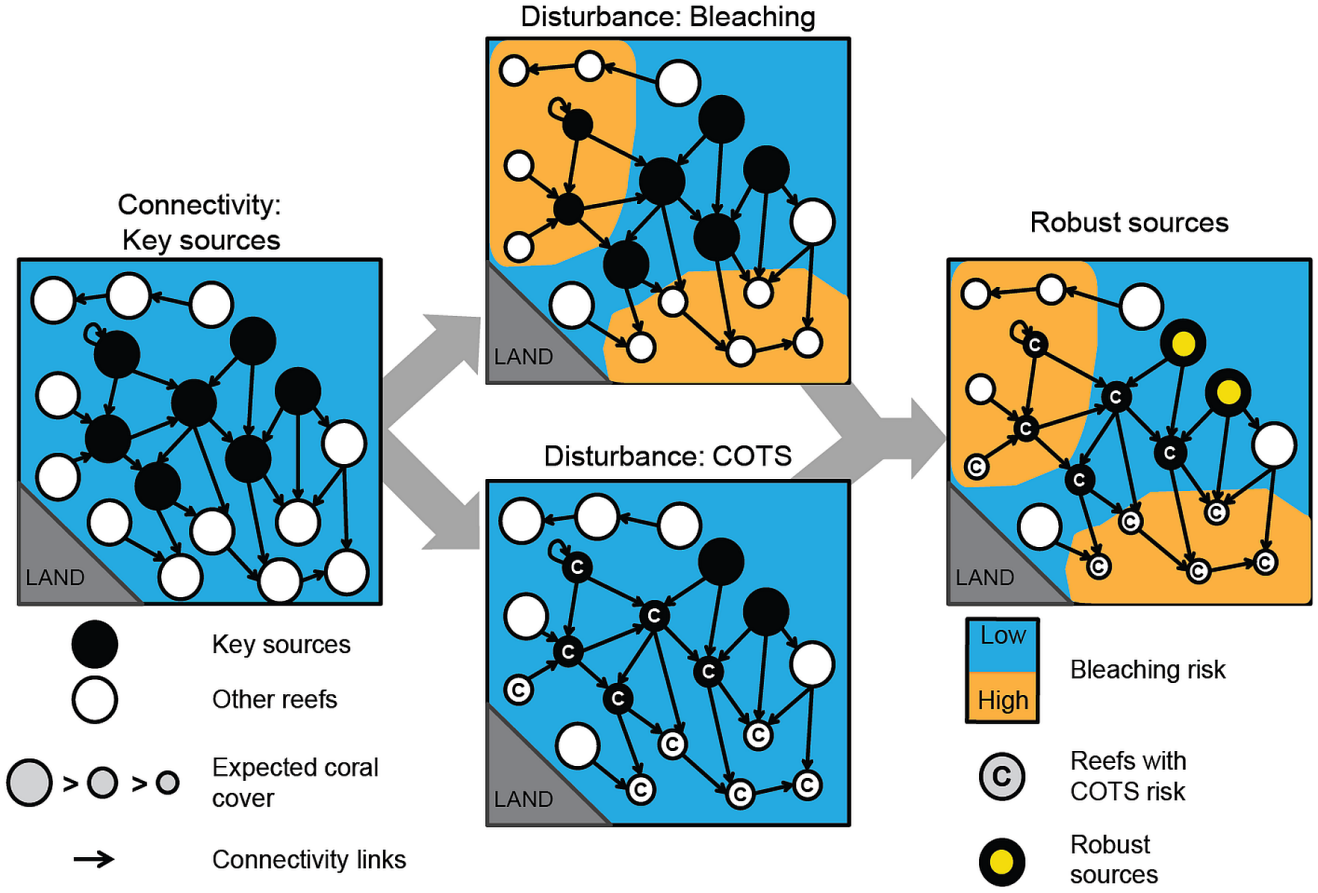
Conceptual diagram describing the process for identifying robust source reefs in a coral reef system. Ocean currents disperse larvae and create interpopulation connectivity links among reefs, leading to an emergence of source reefs with high potential to support coral replenishment. For robust sources, high connectivity will be complemented by a reduced chance of being affected by disturbances, such as thermal stress–induced bleaching, and COTS outbreaks. COTS, crown-of-thorns starfish.

Three criteria would need to be met to maximise the likelihood that a reef would successfully contribute to recovery of the wider ecosystem (Fig 1). First, a source population should be able to supply other populations, including other sources, and thus make an exceptional contribution to the recovery of large portions of the system. Moreover, while demographic connections in marine systems tend to be variable and transient [18], a source should be able to provide consistent replenishment under a variety of oceanographic conditions. Second, a source should exhibit a lower exposure to disturbances so that it can maintain the adult brood stock required to initiate the recovery process of affected areas [19,20]. Third, a source that primarily helps replenishment should distribute desirable organisms but also not distribute undesirable organisms such as pests or invasive species [21]. Simultaneously meeting the first and third requirements is particularly challenging because the connectivity of multiple marine taxa is often highly correlated and driven by the same dispersal mechanisms [22], making high levels of oceanic connectivity a ‘double-edged sword’ that can both help and hinder recovery. Reefs that meet all 3 criteria would confer resilience to a wider coral reef system by facilitating rapid large-scale recovery after major disturbances. However, we are unaware of any study into the relationship between source-sink characteristics of reefs and their exposure to disturbances. Whereas areas of high flow and upwelling have been associated with lower risks of coral bleaching [23], the role of reefs as larval sources is geographically complex, integrating the distribution and sizes of reefs with respect to the speed and, critically, the directions of flow [24]. There is, therefore, no a priori reason to expect that any reefs will meet our 3 desirable criteria in practice. We describe our underlying assumptions about these criteria in the Materials and methods (see also S1 Table).

Here, we explore connectivity and disturbance properties of the world’s largest coral reef system, the Great Barrier Reef (GBR). Despite its size, the combined effects of multiple stressors including coral bleaching [5,16,25,26], cyclones [16,27,28], and outbreaks of corallivorous crown-of-thorns starfish (*Acanthaster sp*., or COTS) [26,29,30] have caused significant declines in coral cover in recent decades [31]. Yet we discover that the GBR also possesses a level of systemic resilience in there being a system of reefs that meet all 3 desirable criteria of reduced disturbance exposure and high recovery potential owing to a common link among oceanography, geography, and connectivity.

## Results

### Criterion 1: Important sources of larvae

**Fig 2:**
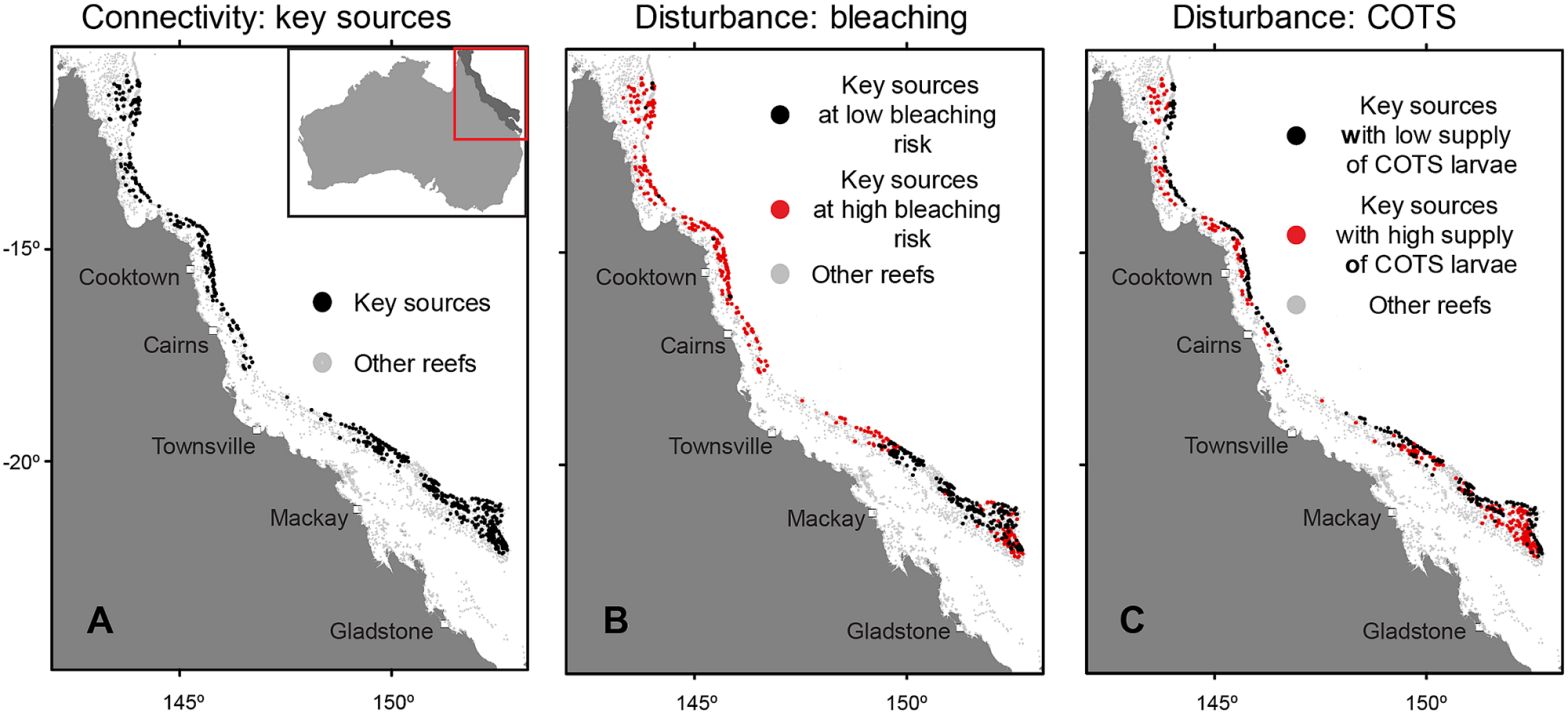
Identifying reefs that exhibit high connectivity and low disturbance exposure. (A) Locations of key source reefs on the GBR with high replenishment potential. (B) Classification of key source reefs according to bleaching risk (>6 DHW) during major bleaching events between 1982 and 2017. (C) Classification of key sources according to their predicted supply of COTS larvae. Data provided in S1 Data. COTS, crown-of-thorns starfish; DHW, degree heating weeks; GBR, Great Barrier Reef.

The connectivity of larvae across the approximately 3,800 reefs of the GBR was modelled using ocean circulation simulations and generated 208 networks, each representing a unique combination of taxa, intra-, and inter-seasonal variability that can influence patterns of dispersal [32–34]. In order to find reefs that satisfy the first criterion of being important sources of replenishment, we examined the resulting connectivity patterns to identify key source reefs that provide consistent replenishment across a range of dispersal conditions and life history strategies (details in the Materials and methods and S2 Table). Each reef’s importance as a source of replenishment was classified using a set of graph-theoretic measures, with the aim of capturing both short-term and long-term outlooks of a reef’s importance to GBR-wide connectivity and prioritising connections that resupply other well-connected sources in the system. Although reefs needed to satisfy an array of connectivity conditions in space and time in order to qualify, we found that 545 reefs (14%) meet the criterion of being strong, consistent sources to a large numbers of reefs, including many major source reefs downstream (Fig 2A). Most of these key sources were located in offshore and mid-shelf regions of the GBR, which is broadly consistent with oceanographic patterns of inflow of oceanic water from the South Equatorial Current [35].

### Criterion 2: Lower thermal stress and exposure to coral bleaching

We then asked which of these key source reefs met our second criterion of naturally lower exposure to acute thermal stress such that their coral populations are likely to remain in relatively good condition even after bleaching events. We focus on corals not only because of their intrinsic importance for biodiversity but because they are the principal engineers of reef habitats [36]. Reefs with healthy coral also harbour higher densities of many vertebrate and invertebrate taxa [37], which increases the potential of such reefs to replenish community diversity.

We examined patterns of thermal stress during all 10 known warming events between 1982 and 2017 (including the most recent bleaching events [5]) using conventional measures of degree heating weeks (DHW). Preliminary observations from the recent GBR bleaching episodes suggest that coral mortality on the GBR starts to occur once thermal stress exceeds 6 DHW (measured in °C–weeks) in a season (personal communication, Mark Eakin to KH). We therefore set a conservative criterion that target reefs would never have experienced >6 DHW during the full 36-year time series. Across the entire GBR, 1,258 reefs (33%) met this condition. Of the 545 ‘key source’ reefs identified under the first criterion, 45% (245) also met the second criterion of being a refuge from thermal stress (Fig 2B).

It is worth noting that while elevated sea temperature is the primary cause of coral bleaching, areas that experience eutrophication can also have an elevated risk of bleaching [38], although this will not necessarily always be the case [5]. Importantly, the offshore location of the identified ‘cool’ source reefs would also limit their exposure to terrestrial runoff [39], which reinforces the notion of low bleaching risk on these reefs.

### Criterion 3: Lower risk of conveying COTS outbreaks

Our third criterion for an ideal source of coral replenishment is that the reef disperses larvae of desirable species (e.g., coral) rather than pests. COTS are the most important pest on the GBR, and their outbreaks are a system-wide problem [26]. Large numbers of adult COTS found during outbreak conditions can strip a reef of its corals [30,40]. Ocean currents then spread the starfish larvae from reefs with ongoing outbreaks, causing large-scale outbreak events which eventually lead to widespread decline in coral [30,31,41]. If a reef important for coral replenishment is exposed to COTS larvae and experiences an outbreak, it could simultaneously lose its value as a source of coral larvae and exacerbate the widespread coral loss by serving as a hub for dispersing COTS larvae. Clearly, such characteristics would be at odds with the role of larval sources important for promoting coral recovery.

**Fig 3:**
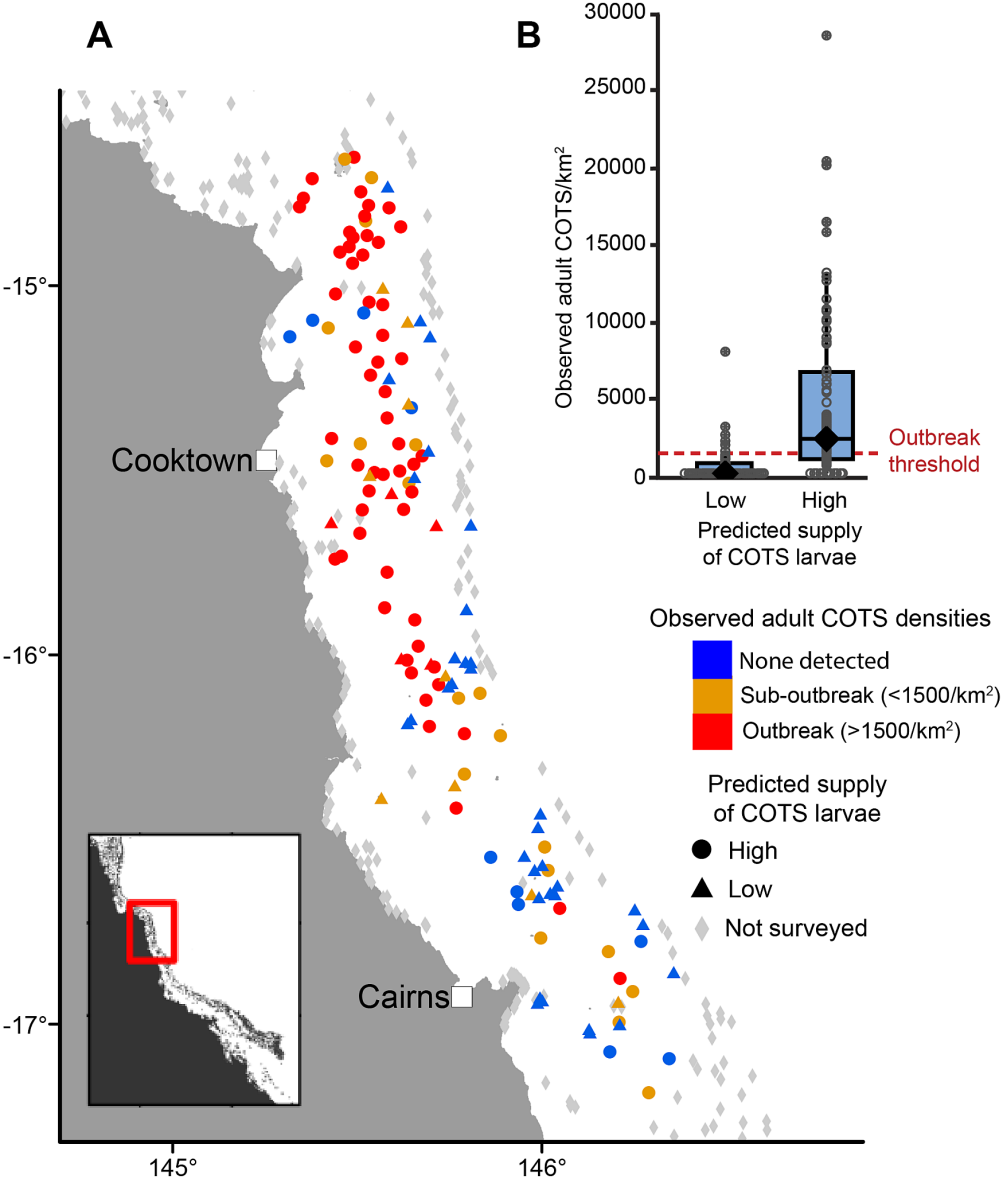
Linking connectivity and COTS abundance estimates from field surveys. (A) A map of the GBR region surveyed for COTS in 2013–2015. Reefs with low predicted supply of COTS larvae (triangles; *N* = 48) were more likely to have low levels of adult COTS or no adult COTS detected (blue symbols). The highest incidence of COTS outbreaks (red) was observed on reefs with high potential supply of COTS larvae (circles; *N* = 89). Note that, although the outbreaks originated in the area north of Cooktown and spread southwards, latitude and longitude were not significant predictors in the analysis, but they were kept in the model as covariates to ensure that any spatial pattern observed in the figure did not affect the observed effect of connectivity. (B) Reefs with high predicted supply of COTS larvae had significantly higher densities of adult COTS. The outbreak threshold of 1,500 adult COTS km^−2^ is shown as a red line [30]. The box plots show medians (black diamonds) and quartiles (blue box). The analysis also included a datum in the high category with an estimated COTS density of >30,000 per km^2^. Data provided in S1 Data. COTS, crown-of-thorns starfish; GBR, Great Barrier Reef.

To assess the risk that potential sources of coral replenishment might also double as harmful sources of COTS larvae, we began by using recent COTS population surveys to validate the predictions of a COTS connectivity model. Because the dispersal simulated in our models (2008–2013) immediately predates the period of intense COTS surveys (2013–2015), reefs predicted to have received more COTS larvae should have higher population densities of adult COTS owing to progressive buildup of COTS populations [30] and have adult COTS densities that imply an active COTS outbreak [42]. We performed a classification analysis contrasting the predicted larval supply against field survey observations to determine the threshold for influx of COTS larvae below which reefs would be unlikely to experience COTS outbreaks (see Materials and methods; S1 Fig). We found that reefs predicted to be consistently in the bottom 30th percentile according to relative larval supply only had an 8% chance of being in an outbreak state. Thus GBR reefs were divided into 2 categories—‘low’ and ‘high’—with respect to the risks of a COTS outbreak linked to relatively high larval supply. As expected, surveyed reefs with high predicted supply of COTS larvae had higher population densities of adult COTS, experiencing on average 4 times higher maximum adult densities (*N* = 137, *t* = 4.6, *p* < 0.0001; Fig 3A and 3B; S3 Table; external import of larvae was the only significant predictor in the model). Also, the odds ratio that reefs with high import of larvae will have outbreaks versus those with low import was 14.6. This validation of our models was also supported by analysis of a second dataset from COTS eradication efforts, which showed the same pattern of higher densities of COTS adults on reefs with high predicted supply of COTS larvae (S4 Table). We conclude that the reefs in the bottom 30% in terms of relative larval supply satisfied the third criterion of having low risk of experiencing COTS outbreaks, and therefore also presented a lower risk of becoming sources of COTS larvae in the system.

Given that our model of COTS dispersal has empirical support, we then evaluated the risk that key source reefs that satisfied the first criterion would become supplied with COTS larvae and experience an outbreak. Despite the fact that key sources were identified on account of their high downstream connectivity for multiple taxa, nearly half (48%, or 262) of them were found to have low upstream connectivity and therefore a low risk of becoming hubs of COTS larvae (Fig 2C). The fact that some key sources also have low upstream connectivity can be attributed to the interaction between their geographic location and oceanography: strong incoming currents originating from the open ocean limit upstream connectivity of outer parts of the reef shelf and therefore also reduce the chance of external colonisation by COTS larvae. Thus, while both COTS and coral larvae rely on the same hydrodynamic forces for dispersal, differences in the supply of COTS larvae lead to a decoupling between the potential sources of both COTS and coral larvae and those more likely to serve as sources of coral larvae alone.

### Integrating connectivity and disturbance patterns

**Fig 4:**
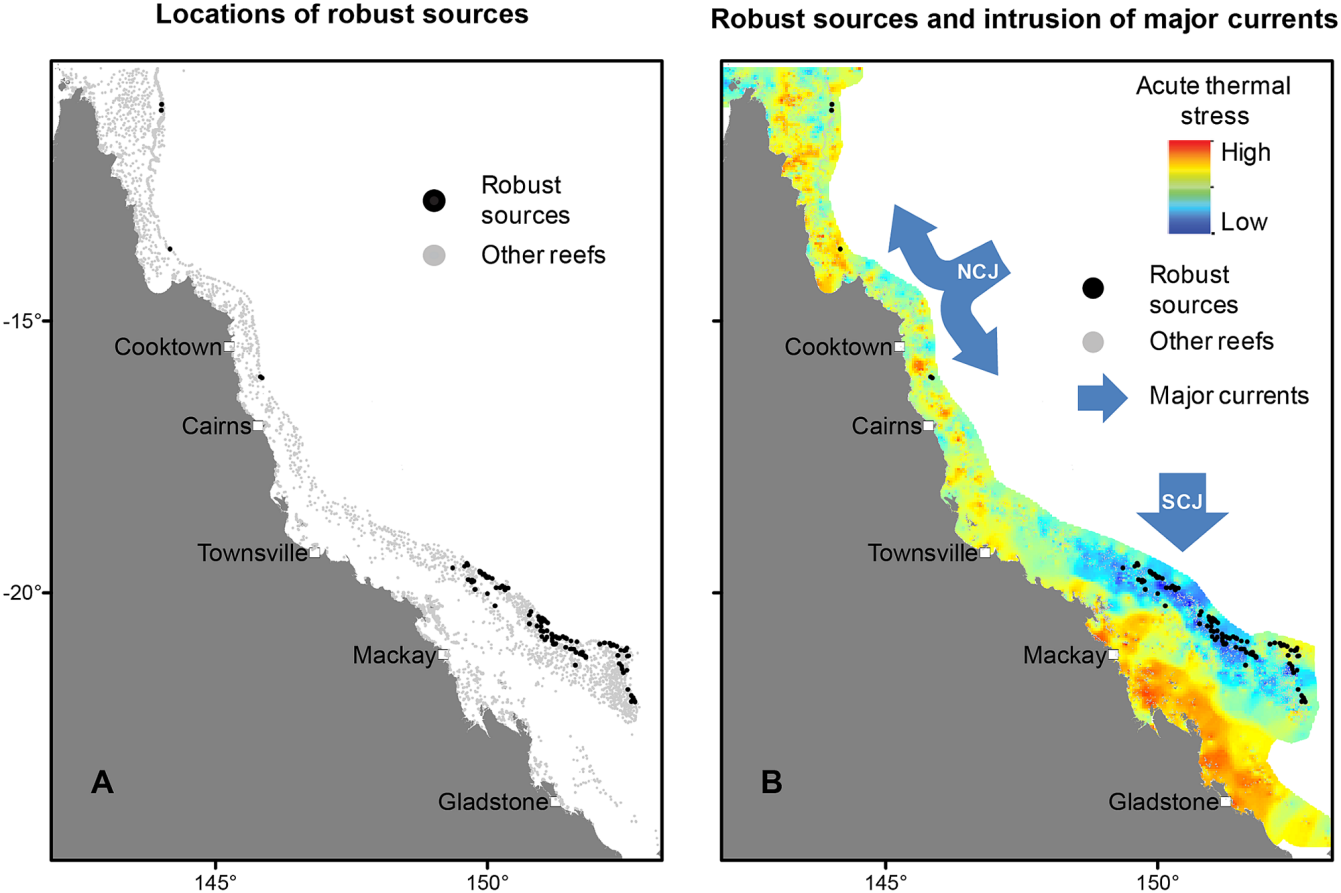
Identifying robust sources on the GBR. (A) Robust sources are the reefs that possess high replenishment potential while also having low risk of bleaching and COTS outbreaks. (B) When robust sources are superimposed on estimates of acute thermal stress, the region of lower stress in the southern GBR is clearly visible. Most robust sources are located in a region where cooler oceanic water of the SCJ, and to a lesser extent the NCJ, of the South Equatorial Current flushes the GBR reef matrix [35]. Data provided in S1 Data. COTS, crown-of-thorns starfish; GBR, Great Barrier Reef; NCJ, North Caledonian Jet; SCJ, South Caledonian Jet.

To qualify as an important source of replenishment even when the system is affected by disturbances, which we term a ‘robust source’, a reef must meet all 3 of the listed desirable criteria. A total of 112 reefs met all criteria (Fig 4A), primarily in outer shelf positions because strong currents from the open ocean bring cooler waters while also facilitating the dispersal of coral larvae landward and avoiding some COTS dispersal problems because of the paucity of upstream reefs (Fig 4B).

While testing for a significant association among the 3 criteria could conceivably be used to evaluate the likelihood of finding this many robust sources on the GBR, the presence of spatial autocorrelation within each data layer (key sources of coral larvae, thermal stress, and risk of COTS) as well as among data layers makes it unclear whether statistically robust conclusions could be drawn from such analyses. Moreover, since every reef in the GBR was evaluated for the 3 criteria, the benefits of a statistical test that would use random subsamples of reefs to determine the null likelihood of a reef meeting all 3 criteria are unclear even if a test could be devised to account for the difficult problems of spatial autocorrelation. As such, rather than evaluating the significance of finding a certain number of robust sources, we instead evaluated their potential importance for supplying larvae to the wider GBR.

**Fig 5:**
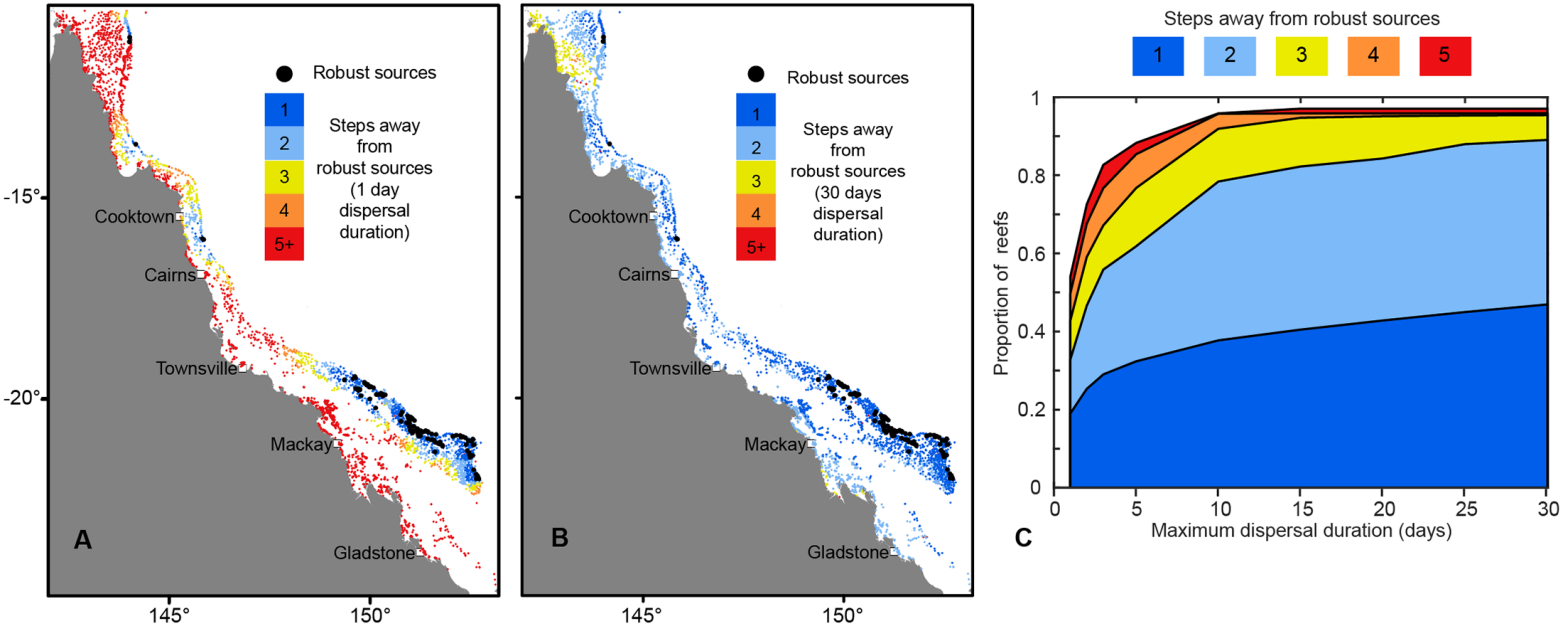
Distance of reefs on the GBR from robust sources in terms of colonisation steps. (A) Number of colonisation steps needed to reach reefs from robust sources after 1 day of dispersal. (B) Number of colonisation steps needed to reach reefs from robust sources after 30 days of dispersal. (C) Percentage of reefs that can be reached in a given number of consecutive colonisation steps as a function of maximum dispersal duration (measured in days since release). Values for a single colonisation step correspond to direct links. After 30 days of dispersal, >80% of the reefs were within 2, and >95% of the reefs were within 5, colonisation steps away from robust sources. Data provided in S1 Data. GBR, Great Barrier Reef.

Although robust sources comprise a small proportion (3%) of the GBR, they can supply around 19% of all reefs after a single reproductive event if the larval duration is short (Fig 5A, 1 day), increasing to 47% of reefs for longer larval durations (Fig 5B, 30 days). Such high scalability reflects the value of using stringent connectivity criteria in the site selection process. These estimates of potential impact (supplying 19%–47% of reefs) only consider direct connections from sources during a single dispersal event, and direct replenishment would amplify over time as the coral metapopulation recovers (Fig 5B and 5C). If we consider the importance of replenishment over successive colonisation steps following a stepping-stone pattern, then the number of reefs benefiting from robust sources escalates rapidly (Fig 5). For example, with a maximum larval duration of 30 days, >80% of all GBR reefs were within 2 colonisation steps from robust sources, and >95% were within 5 colonisation steps (Fig 5C). While these numbers will be contingent on how long the larvae can survive during dispersal, once larval survival exceeded 10 days, the pattern of replenishment was fairly consistent up to maximum investigated survival duration of 1 month (Fig 5C).

It should be noted that this analysis describes the best-case recovery scenario in which corals are able to colonise sink reefs while also assuming that there are no system-wide disturbances between successive colonisation steps to impede regional recovery. While the recovery dynamics will inevitably be more complex than this and foster spatial heterogeneity in recovery rates, consistently high connectivity of robust sources across multiple spawning seasons and life history traits makes them the most likely candidates to fulfil this role in the system.

## Discussion

While the GBR benefits from one of the most ambitious sets of no-take reserves for coral reefs [43], we find that it also has an inherent level of systemic resilience: a set of robust source reefs that are positioned to facilitate processes of coral recovery throughout much of the wider ecosystem. This list of around 100 reefs is both a tangible and feasible set of intervention points to form part of a strategy for maintaining the systemic resilience of an ecosystem that is thousands of kilometres in scale. While the presence of such reefs on the GBR is encouraging, the fact that only 3% of the reefs meet all 3 criteria underlines the need for effective local protection and reduction of global stressors in order to support their ongoing role in the ecosystem. Given that larval transport and thermal bleaching are important characteristics of coral reef ecosystems worldwide, similar synergistic effects of ocean circulation are likely to be observed in other coral reef systems.

A potential drawback of high flow and advection at source reefs is a reduction in the level of larval retention. Indeed, local retention in 80% of robust sources was lower than the GBR median (S2 Fig). Whether such reductions might compromise the recovery rate of robust sources is unclear, although their relatively low exposure to thermal stress implies that recovery would be required infrequently.

Interestingly, robust sources seem to have relatively little exposure to another major source of disturbance: cyclones. The spatial distribution of robust sources shows little congruence with cyclone risk [28], which is greatest in the central GBR where the density of robust sources is low (S3 Fig). This implies, firstly, that the term ‘robust sources’ as used here is relevant in the context of cyclone disturbance as well as thermal stress. Secondly, while regions experiencing frequent cyclone damage are associated with fewer robust sources, it is important to bear in mind the large-scale colonisation potential of robust sources for the wider coral reef network, in which only 3% of reefs have the potential to supply 15 times as many reefs in a single dispersal event (Fig 5). Thus, while stochastic cyclones will inevitably ‘decommission’ several robust sources at any given time, the geographic spread and strength of the network should help mitigate such impacts.

Although robust sources have been identified across the GBR, a distinct cluster occurs in the south. When considering the combined spatial patterns of exposures to multiple stressors, the offshore parts of the southern GBR appear to be a regional refuge, notably from recent bleaching and COTS outbreaks that primarily affected the northern regions [5,30], as well as from cyclones (S3 Fig). That this region also possesses many robust sources implies that the southern GBR is likely to be exceptionally resilient and also positioned to stimulate recovery elsewhere (Fig 5). Although not explicitly considered here, this region is also less likely to be affected by local anthropogenic impacts such as decreased water quality caused by agricultural runoffs from rivers, as it tends to be located far offshore where the effects of poor water quality should be less pronounced [39,44–47]. However, global-scale stressors such as changes in carbonate chemistry owing to ocean acidification [48,49] or changes in ocean current patterns [50,51] might yet have a notable effect on the GBR’s systemic resilience.

We build on the applications of graph theory in conservation and the role of connectivity in metapopulation persistence. A key focus in many literature examples has been to identify metapopulation network robustness to random perturbations and the identification of cut-nodes that maintain system coherence [33,52–57]. We extend these approaches by proposing specific sites of importance based on spatial heterogeneity in both connection strength and stress rather than on random perturbations. Another application of graph theory and connectivity has been to disrupt the spread of pests, pathogens, and invasive species [41,58–62]. We do not address the question of how to disrupt COTS here, but future studies might provide an integrated approach that attempts to optimize the maintenance of system recovery (this paper) while simultaneously attempting to disrupt pests like COTS. In the meantime, it would be prudent to prioritise protection from anthropogenic and other manageable stressors, including fishing [63,64], anchoring, and COTS to ensure that the resident coral populations on robust sources continue to play a role in system replenishment. Fortunately, robust sources are already overrepresented in the current set of no-take zones of the GBR: the current zoning plan [65], which affords no-take status to 33% of reef area and 27% of individual reefs, includes 46% of all robust sources in its no-take regions (S4 Fig).

The approach described here attempts to operationalize the idea of systemic resilience in a marine system [17], as failures in recruitment may result in a cascade of failures across the system [66]. The science of systemic resilience is fairly young and lacks firm guidelines; here, we opted to use stringent criteria that resulted in 3% of reefs being designated as robust sources. Yet, many reefs will remain moderately functional even if they fail to meet our strict criteria [4,25,27]. Thus, future work will consider how systemic resilience attenuates as the underlying criteria are relaxed. Equally, estimates of systemic resilience will need to consider additional stressors, including water quality, biogeochemistry, and differences in key ecosystem processes (e.g., herbivory) and reef-level coral community composition as such data become available and integrated with ecological models.

Our approach implicitly assumes that coral larvae emanating from reefs that experience relatively low thermal stress are able to replenish populations subjected to higher thermal stress. This assumption has never been tested formally, though it seems reasonable. Supporting evidence includes the fact that coral populations disperse over broad thermal environments on both ecological [12] and evolutionary [67] scales and that juvenile corals appear to be particularly robust to thermal stress [68]. Moreover, transgenerational plasticity may generate hardier offspring, providing that corals can survive long enough to reproduce [69]. Yet, contrary mechanisms might include genetic adaption to lower stress levels in robust sources [70] and genetic homogenization as total population size declines [71].

That we identified a series of reefs that appear to play a disproportionately important role in driving regional recovery does not imply that the GBR, and its robust sources, will be immune to future disturbances. For example, while robust sources have had demonstrably lower risk of thermal stress over the past 36 years, the efficacy of their role will likely change in the future. The GBR has recently experienced major bleaching events over 2 consecutive years (southern hemisphere summers of 2015–2016 and 2016–2017), the first of which had an unprecedented spatial pattern, severely affecting the northern reef for the first time [5]. Indeed, the paucity of robust sources in this region reflects the impact of the anomalous 2016 bleaching event. Thus, a key objective for future analyses is to estimate the spatial patterns and return times of major bleaching events and their potential impact on the functioning of the GBR as a resilient system. This is a daunting challenge, as it requires careful downscaling of global climate models that are currently unable to resolve meaningful spatial patterns of future warming on a GBR scale [72].

Validating predictions of systemic resilience is challenging. Formal tests would require simultaneous data on the dynamics of multiple source-sink reefs, yet less than 3% of reefs are actively monitored (albeit, in the largest monitoring effort of any coral reef system). Rather, we must rely on the efficacy of the inputs—each of which have experienced some level of testing in their own right—and the conservative way in which criteria were applied (see also S1 Table). Firstly, while there has never been an empirical demonstration of the ability of a coral connectivity model to predict demographic effects and realized larval supply, there have been a number of studies to test the predictions of oceanographic particle dispersal models for predicting coral gene flow [67,73], which found high—albeit imperfect—levels of congruence between models and population data, especially in identifying areas of restricted gene flow. Secondly, DHW has been a widely used metric for exploring the effects of thermal stress on coral assemblages and, although low levels of DHW may be associated with bleaching, they tend to elicit limited coral mortality [4,74–76]. There is, however, scope to improve the functioning of algorithms to predict the likelihood of coral mortality, such as including the effects of solar radiation and recent thermal trajectories [77]. Finally, in this paper we evaluated the efficacy of connectivity models to predict COTS dispersal dynamics though comparisons with field observations of emergent COTS outbreaks. We point out that advances in the use of machine learning and other tools (e.g., semiautomated image analysis, especially when combined with citizen science initiatives [78]), will likely provide a greater opportunity to test model projections in the future.

Our discovery of systemic resilience in the form of reefs with high recovery potential complements another recently discovered resilience mechanism: pulses of warm water that precede major thermal stress and help prepare corals to tolerate heat stress and diminish the impacts of bleaching [77]. Although the protective role of prewarming pulses is projected to weaken and possibly even disappear under business-as-usual greenhouse gas emissions, robust sources are likely to be some of the most persistent sources of replenishment because of their low susceptibility to warming events. Yet, uncertainty about disturbance patterns means that the importance of mitigating greenhouse gas emissions remains vital for ensuring prolific corals in the GBR’s future [36]. Furthermore, since reef recovery is not only driven by larval supply, postsettlement processes at sinks may strongly determine successful recruitment and growth [79,80]. Therefore, local practices to improve water quality [45–47], stabilise rubble [81], and avoid ecosystem overfishing of herbivores [63,64,80], as well as global initiatives to reduce the ultimate burdens on reefs, such as human impacts on the environment and climate [71,82,83], all have a role to play in assisting successful coral recovery. The importance of supporting such natural recovery processes will likely increase in the future as climate change reduces the average size of coral populations and the need for recolonization becomes more frequent.

## Materials and methods

### Design of dispersal and connectivity models

To obtain patterns of population connectivity across the GBR, Lagrangian dispersal simulations were performed using *Connie2*, a high-resolution advection/diffusion oceanographic model of the entire GBR region. The technical aspects of the hydrodynamic dispersal model have been previously described in detail and published elsewhere (see also www.csiro.au/connie2/ for a web interface) [41,84]. In the simulations, individual reefs of the GBR were represented as the convex polygons that encompassed all GIS coordinates that define the actual GBR reefs [41]. To account for inter- and intra-seasonal differences in oceanographic circulation, dispersal was simulated for 16 distinct spawning events (4 spawning events per summer, i.e., December to March, for 2008–2009, 2010–2011, 2011–2012, and 2012–2013). To obtain a connectivity network for a specific spawning event, 10 dispersal simulations of 10^3^ particles each were run for each of the 3,806 reef polygons. Spatial displacement of particles was resolved in hourly intervals using the fourth-order Runge-Kutta scheme that advected the particles across raster grid with 4 km resolution of oceanographic forces. Particles were dispersed passively by the oceanographic forces (complex swimming or homing behaviours were not explicitly modelled) and were also considered to be negatively buoyant (dispersed at a constant depth of 0.5 m; although particles remained negatively buoyant during dispersal, the underlying oceanographic model included 3-dimensional representation of currents, e.g., upwelling, that could affect horizontal displacement of particles). This dispersal modelling framework has previously been successfully employed to infer COTS dispersal patterns on the GBR [41,61]. The current set of simulations was substantially expanded to cover a much broader range of dispersal characteristics and spawning events.

While the majority of connectivity models have focused on static connectivity relationships, in many ecosystems where dispersal relies on ephemeral forces, such as marine larvae dispersed by ocean currents, connectivity tends to be highly variable and transient [18]. In order to identify sources that help with recovery of the wide range of coral reef organisms and conditions, we designed our models to be inclusive of a wide range of life history characteristics. Larval survival and competency can be affected by numerous factors, many of which have only been ascertained in laboratory conditions and may be very different in the field [22,34]. Even within a taxon, dispersal potential can vary both between and within seasons with parameters such as temperature and nutrient availability but also due to more ephemeral conditions like river outflows that dynamically affect larval survival and competency [29,34]. Using a fixed set of parameters for each species therefore means obtaining a connectivity pattern of a species that may only represent dispersal patterns under a specific set of conditions while ignoring all other possible combinations. To address these complexities with our models, we opted to base our analyses on a wide range of competency curves and spawning times rather than use a few parameter combinations to explicitly characterise individual species (as noted later, we used a certain range of these parameters, rather than specific values, for COTS).

Gamma functions were used to model larval competency (all scale parameters equal to 1; see S2 Table for shape parameters). Three different daily mortality rates (constant rates of 0.05, 0.1, and 0.2) were tested to cover a range of empirically determined values for invertebrate larvae [85]. Since most of the connectivity measures were relative (for example, determining whether a reef had stronger or weaker connectivity links than average), connectivity results were found to be robust for the range of mortality rates, with the same reefs being identified as key sources in 95% of the cases. Mortality rate of 0.1/day was used in the presented analyses. Parameter values for larval competency and mortality were then combined to define 13 different survival-competency curves for each of the 16 spawning events. These curves were diverse enough to represent life histories of different organisms, from those whose larvae only remain competent for a few days to those whose larvae can spend weeks in the water column.

Inclusion of competency and mortality made it possible to treat the individual particles not as individual larvae, but rather as a pool of larvae that were competent and surviving at any point in time that could disperse from the source along the recorded trajectory. The proximity of particles to reefs was checked every 12 hours after release. When a particle was located <1 km away from a reef polygon, it was considered to be ‘arrested’ by the reef. Particles arrested by a reef did not move any further and instead contributed to the connectivity between reefs proportionally to the amount of larvae determined to be competent and surviving at the time of arrival. This process continued until the maximum number of days the larvae could survive during dispersal (see S2 Table). Arresting the particles in proximity of reefs also served as a simple proxy for any potential fine-scale hydrodynamics around reefs or short-range homing behaviours [86–88] and thereby ensured that the reefs were treated as physical obstacles to dispersal.

A combination of competency, survival, and seascape characteristics meant that only about 0.0002% of the larvae in our models settled after day 25. While such low levels of settling larvae as those observed during the later stages of the simulations could be detected with methods examining genetic connectivity and allelic composition of populations [67], they are unlikely to have a major effect on replenishment of populations after disturbances when many more larvae are generally needed to produce a demographic effect. Although some organisms, including some coral species, are known to have larvae that can survive in the water column for extended periods of time, the focus on demographically relevant connectivity meant that the maximum period during which the larvae could settle in our simulations was therefore limited to 30 days (see S2 Table).

The relative contribution from a specific source reef to a specific sink reef was then used as the strength of a connection between these reefs in a connectivity network. The information on the asymmetry in source-sink exchanges was retained in the networks, resulting in all connectivity networks being directed (digraphs). A set of 208 directed connectivity networks was obtained by combining multiple spawnings and competency curves. Taken together, these connectivity networks were therefore not only capable of representing dispersal of a broad spectrum of coral reef organisms using different spawning regimes and life history strategies but could also emulate cases when local conditions would alter the dispersal pattern of a taxon [29,34]. Consistency in connectivity relationships was then used to determine which connectivity links were less likely to be affected by the inherent transience of marine dispersal and empirical uncertainty surrounding dispersal parameters.

### Connectivity network analysis

A broad spectrum of connectivity patterns represented by the connectivity networks was used to identify key sources that can facilitate the recovery of a wide range of coral reef organisms. Since different graph theory metrics can capture different aspects of source reef’s replenishment potential, 5 properties were identified in each connectivity network to make the analysis comprehensive (graph theory analogues provided in parentheses): (1) number of reefs a source supplied (node’s out-degree), (2) total amount of supply a source provided (node’s strength), (3) number of links through which a source provided more than 10% of the relative supply to a sink (node’s out-degree when considering only links that provide >10% of supply to a respective sink) [22], (4) number of other sources a source could supply (node’s out-degree when counting only links to major sources that were identified by using the properties 1, 2, and 3), and (5) the number of other reefs in a network that could be reached via a directed path from a source (node’s out-component) [41]. These 5 connectivity properties were measured for all reefs and across 208 scenarios representing multiple life-history characteristics and seasons of dispersal. There are many ways in which these data could be used to identify ‘key larval sources’, and our decision reflected certainty over their importance. Although a reef could in theory be considered ‘well connected’ if it only satisfied 4 of the specified criteria, we wanted the identified reefs to exhibit high potential for both short-term and long-term recovery as well as supporting a recovery ‘cascade’ by supplying other sources. As such, we wanted to be strict in that a reef needed to exhibit all 5 connectivity criteria to meet our aim of being an effective source that is also connected to other major sources, thereby maximising the rate at which replenishment can occur across the reef system. We therefore specified that all 5 connectivity criteria must always be met. This left 2 other decisions: how much should a reef excel within any individual connectivity metric (i.e., where does it rank within the 3,806 GBR reefs?), and over how many of the possible scenarios should it excel? Currently, there is no empirical demographic justification available for selecting a critical threshold because rigorous testing of larval dispersal models for predicting rates of realised larval supply in the field has never been undertaken anywhere for logistical reasons. Therefore, we specified a somewhat arbitrary threshold in that a reef must have scored above average (top 50th percentile) in all 5 connectivity metrics and do so in above average (top 50th percentile) number of scenarios in order to qualify as having high potential for recovery of the system. Implementing these criteria served as a strong filter as only 14% of reefs succeeded in being designated as ‘key sources’.

Since demographically relevant levels of connectivity do not tend to occur over the entire length of the GBR, the ranking of the reefs according to their replenishment potential was performed within the natural resource management areas that are used to manage the GBR by the GBR Marine Park Authority [65].

In order to estimate a reef’s potential to be supplied by other reefs or through local retention of larvae, we also determined the relative amount of supply a reef received from other reefs as well as the relative level of local retention of larvae (the amount of settled larvae for which the destination reef was the same as the source reef). External supply was used to determine the predicted supply of COTS larvae to the reef. Reefs that had low potential of local retention in most of the networks could therefore exhibit a reduced potential for recovery. Low local retention of larvae could also be indicative of high levels of flushing and different flow regimes experienced by reefs (S3 Fig).

### Thermal stress analysis

Mass coral bleaching has been shown to be caused by prolonged periods of thermal stress which is typically expressed using the DHW metric. DHW is a cumulative measurement of the intensity and duration of acute thermal stress and is expressed in the unit °C-weeks. Here, we used 2 satellite sea surface temperature datasets to estimate maximum annual (1982–2017) DHW across the GBR: For the years 1982–2012, DHW was calculated using Version 5 of the Coral Reef Temperature Anomaly Database (CoRTAD) [75], a weekly 4 km product. CoRTAD DHW was derived using the methods adopted by NOAA Coral Reef Watch that accumulates any hot spots >1°C over a 12-week window. For years 2013 to 2017, we used ReefTemp Next Generation (RTNG), a high resolution (0.02°) daily product developed by the Australian Government’s Bureau of Meteorology as a key component of the Great Barrier Reef Marine Park Authority’s (GBRPMA’s) Early Warning System [89].

The maximum annual DHW was extracted for each of the pixels from the 2 datasets that intersected GBR reef polygons, corresponding to 5,059 pixels from CoRTAD and 14,324 from RTNG. Next, we defined significant thermal stress events as those years where >50% of all reef pixels experienced DHW > 0, which occurred in 10 summers (1982, 1986, 1987, 1992, 1998, 2002, 2010, 2011, 2016, and 2017) of the 36-year time series. Finally, thermal stress refugia reefs (*N* = 1,258) were defined as those reefs that did not experience a DHW > 6 across 75% of their area during those same 10 years. In other words, ≥75% of the reef area did not experience thermal stress associated with bleaching mortality. Although the threshold above which some reefs will bleach is usually taken to be 4 DHW [4,23,74], during the recent widespread bleaching events on the GBR, significant mortality from bleaching was observed above 6 DHW threshold (personal communication, Mark Eakin to KH).

To estimate the relative levels of thermal stress over time, reef polygons were also ranked by their mean DHW over the same years. These rankings were standardized between 100 (highest DHW) and 1 (lowest DHW), providing a thermal stress ranking index that was used to illustrate the correspondence between the relative levels of acute thermal stress and the approximate locations of major ocean currents that flush the GBR in Fig 4B.

### COTS spatial dynamics and analysis of field surveys

COTS is a corallivorous asteroid native to the GBR whose rapid increase in numbers can lead to reef-damaging outbreaks that can lead to >90% local coral mortality [30]. The GBR undergoes a major spate of COTS outbreaks every 14–17 years. First outbreaks in such large-scale events tend to appear in the Cooktown-Cairns region of the northern GBR (see Fig 3A for the most recent case and the reefs surveyed in response). Outbreak initiation is most likely preceded by a local buildup of larvae [30] and possibly also augmented by favourable nutrient conditions that enhance larval survival [29]. Once initiated, COTS outbreaks then spread through larval transport and eventually end up affecting large portions of the GBR, with prominent effects on the overall health of the ecosystem [31,90]. As of this writing, the GBR is in the middle of another major COTS outbreak event, with an escalating number of outbreaks since the early 2010s.

While the larval transport is crucial for the widespread impacts of COTS, the exact parameters of life history traits that define the dispersal of COTS larvae are not only uncertain but also known to vary with local conditions such as temperature and nutrient availability [22,30,34]. In laboratory studies, COTS larvae typically become competent at around 9–11 days, but this period can also be extended in conditions of nutrient scarcity or shortened due to nutrient abundance [30,34]. COTS are also known to spawn several times during the GBR summer months, but with no established regularity in timing of the spawning events [30]. To account for these uncertainties and ensure that the simulations can capture the entire spectrum of possible developmental conditions, we approximated COTS larval supply using an entire range of the simulated networks. Reefs were then classified according to whether they consistently had a high or low supply of COTS larvae in more than half of the 16 simulated spawning events that occurred during the 4 years before the field surveys began. With only 2 categories (high or low predicted supply of COTS larvae, allowing ties), a total of 1,904 reefs had been classified to have low risk of being supplied with COTS larvae.

Extensive field surveys performed in response to the ongoing series of outbreaks made it possible to validate the predictions of high-resolution connectivity models with relevant estimates of local population abundances from the field. The first of these surveys were undertaken in 2013; however, COTS populations detected at that time probably underwent a period of buildup for several years before that [30]. Modelling connectivity of COTS larvae during 2008–2013, which immediately predates the field surveys of COTS adults, therefore allowed us to estimate levels of larval transport that would have taken place during that buildup period.

Surveys to estimate the COTS numbers and outbreak locations were performed using a manta tow technique designed for rapid broad-scale surveys of COTS populations, in which towed divers visually assess adult COTS numbers [30,42]. COTS population densities of around 1,500 individuals/km^2^ were found to have damaging effects on coral cover and are used operationally as an outbreak threshold by both scientists and managers [30]. The manta tow survey data have been provided by the GBRMPA.

### Classification of reefs according to outbreak risk

We evaluated the performance of different percentiles of relative larval supply with respect to their ability to dichotomously classify risk of COTS outbreaks on reefs. For this, we first ranked the surveyed reefs for each of the 208 networks in terms of the larval supply they received from other reefs. We then classified reefs into 2 groups according to whether they were ranked above or below a certain percentile threshold for a given network. We tested 19 percentile thresholds, from 5% to 95% in 5% intervals. Reefs that exceeded the specific threshold in an above-average number of scenarios (top 50th percentile of observed maximum) were then classified as having ‘high’ supply for that threshold and the rest as having ‘low’ supply. Reefs with high supply were predicted to have a higher risk of outbreaks, as they consistently received more larvae across a range of environmental conditions and possible life histories.

We then evaluated how well each of the percentile thresholds performed in terms of correctly identifying reefs that were determined to have adult COTS population densities below the outbreak level of 1,500 individuals per km^2^ in field surveys. Of the 137 surveyed reefs, 61 of them (44.5%) had outbreak densities of adult COTS. After comparing the performance of the classification analysis for different larval supply thresholds [91], we found that only 8.2% of the reefs consistently ranked in the bottom 30th percentile of larval supply had outbreak densities of adult COTS (S1 Fig). Based on this result, the 30th percentile was used as a threshold that could reliably identify reefs that are unlikely to have a high supply of COTS larvae and therefore exhibit a lower risk of both experiencing COTS outbreaks and spreading the COTS larvae to other reefs.

### Analysis of the CPUE data from COTS eradication efforts

To supplement the results of COTS surveys, a second set of analysis was also performed using catch-per-unit effort (CPUE) values from eradication efforts undertaken between 2013 and 2015 by the Association of Marine Park Tourism Operators (AMPTO) aimed at controlling the levels of adult COTS on reefs. Local eradication efforts have been organised and implemented in response to the rising levels of COTS. While the eradication efforts have been implemented in approximately the same region as the manta tow surveys, they were not performed on the same set of reefs (though there is overlap). In contrast to the rapid broad-scale surveys of large areas characteristic of manta tows, eradication efforts used intensive search dives by trained divers to locate adult COTS over a small area. CPUE rates obtained during these dives, especially during an initial visit of the eradication team to a reef, can be used as an estimate of the densities of adult COTS present on a section of a reef; although, unlike for manta tows, no firm threshold exists with regard to the expected CPUEs for outbreak versus nonoutbreak conditions. These density estimates can then be used to evaluate the predictors of COTS larval supply obtained from the connectivity networks. The dataset has been provided by AMPTO.

The potential to receive COTS larvae was again an important predictor of CPUE rates for adult COTS on individual reefs, with significantly higher CPUEs on reefs predicted to have experienced a higher supply of COTS larvae (those above 30th percentile threshold) in the years predating the eradication efforts (*N* = 94, *F* = 6.92, *p* = .01). Moreover, external supply of COTS larvae was the only significant predictor in the statistical model. Test details are provided in S4 Table.

The importance of external supply of COTS as a predictor of subsequent densities of adult COTS was therefore corroborated by 2 separate analyses that used 2 independent datasets derived from different methods to estimate adult COTS numbers on reefs.

### Key assumptions regarding 3 criteria for reefs to become robust sources

Our 3 criteria involve a number of assumptions that should be made explicit, even if they are not particularly controversial. The main assumptions are listed in S1 Table.

### Estimating replenishment potential of the robust sources

To assess the potential of robust sources to replenish other parts of the GBR, we have determined the proportion of the GBR that can be supplied by such sources in an average year. We only considered reefs that were directly supplied from robust sources; also, reefs that were supplied from more than 1 robust source, e.g., in areas where robust sources were close to each other, were only counted once. We further considered how many of the identified key sources were supplied per year in order to emphasize the importance of the supply from robust sources on the wider supply cascade. In the context of replenishment and recolonization of disturbed populations, space and density limitations at sinks should be a minor issue and even small levels of supply could end up being locally important. As such, these analyses took into account links of all strengths (that is, we did not impose an arbitrary demographic threshold on minimum link strength). Since the number of reefs that can be supplied from robust sources will also increase with longer dispersal times, we have performed these calculations for all of the simulated larval survival durations (see S2 Table). The results of the analyses are presented in Fig 5.

We have also examined the relative distance of all reefs on the GBR from the robust sources in terms of the number of colonisation steps/stepping stones needed to reach them. This was determined by identifying the directed shortest path from each of the robust sources to all other reefs and then finding the directed shortest path with the fewest number of colonisation steps/stepping stones from any robust source to every other reef on the GBR [57]. The results of this analysis for different larval durations listed (see S2 Table) are shown in different panels of Fig 5.

### Statistical analyses

A linear model was used to determine whether predicted supply of COTS larvae during a putative buildup period can be used to explain the population densities of adult COTS later observed on reefs during field surveys (*N* of reefs surveyed = 137). Densities of adult COTS observed during a survey of each reef when maximum average levels of COTS were recorded per manta tow were used as field estimates of COTS abundance. The geographical locations of the individual GBR reefs were represented by the longitude and latitude of the centroids of reef polygons. Observed coral cover was included as a covariate due to its potential effect on observing COTS in manta tow surveys. Because COTS were not observed on many reefs, a Tweedie distribution with a dispersion parameter of 3.58 was fitted to the data using the *tweedie* package for the *R* platform (https://cran.r-project.org/web/packages/tweedie/index.html) and used to model the zero-inflated frequency distribution of observed COTS densities [92]. The linear part of the model included 7 fixed factors. The factors included 5 continuous predictors: latitude, longitude, date of survey, observed coral cover, and reef size; and 2 categorical predictors: predicted supply of COTS larvae from external sources and local retention of COTS larvae with 2 levels for each (‘high’ and ‘low’) based on the relative rank in COTS connectivity networks. The interaction between the 2 categorical predictors was also considered, but was not significant. The analysis was then performed using the *GLM* package for the R platform. Test details are provided in S3 Table.

A linear model was used to compare the predicted connectivity of COTS with the CPUE values for adult COTS from the eradication efforts. The factors included 5 continuous predictors: latitude, longitude, date of cull, observed coral cover, and reef size; and 2 categorical predictors: predicted supply of COTS larvae from external sources and local retention of COTS larvae with 2 levels for each (‘high’ and ‘low’) based on the relative rank in respective connectivity networks. Unlike manta tows during which many surveys did not observe adult COTS on reefs, in nearly all cases at least some COTS were culled, resulting in a dataset with few zeros, so a generalized linear model was used in the analysis. Since the culls were performed using different boats that hosted the divers, boat ID was included in the linear model as a random factor. Date of cull was added as COTS densities are likely to increase over time. Observed coral cover was included as a covariate due to its potential effect on observing COTS during culls. Due to the potential effect of previous eradication efforts on local COTS population densities, only the CPUE recorded on a first visit to a reef by the divers was used in the analysis. The interaction between the 2 categorical predictors was also considered but was not significant. Test details are provided in S4 Table.

## Supporting information

S1 Fig Evaluating performance of different larval supply thresholds for predicting adult COTS densities in surveys. The threshold of 30th percentile was found to perform the best as it gives the lowest rate of false negatives (reefs that are classified as low risk due to low supply of larvae but also had adult COTS outbreaks in the surveys). This percentile was then used as a threshold to classify reefs that will have low risk of COTS outbreaks. COTS, crown-of-thorns starfish.
(TIF)

S2 Fig Classification of robust sources with respect to local retention of larvae. Black circles represent robust sources that have had consistently high local retention of larvae in dispersal simulations when compared to the GBR-wide average; red circles, well represented in the outer shelf regions, represent robust sources that have had below average local retention levels. The majority (80%) of the robust sources have low levels of local retention, possibly due to high flushing regimes. GBR, Great Barrier Reef.
(TIF)

S3 Fig Locations of robust sources and expected annual frequency of category ≥1 cyclones on the GBR. Robust sources tend to be located outside of the regions with high expected cyclone frequency (coloured background; adapted from data presented in Wolff et al. [28]). GBR, Great Barrier Reef.
(TIF)

S4 Fig Classification of robust sources with respect to their placement in no-take zones. Black circles represent robust sources located in no-take zones; red circles represent robust sources that are not located in no-take zones. Nearly half (46%) of the robust sources are already located in no-take zones and awarded the highest level of protection under the current GBR zoning plan [65]. GBR, Great Barrier Reef.
(TIF)

S1 Table Key assumptions and rationales behind the 3 criteria used to identify robust sources. COTS, crown-of-thorns starfish; DHW, degree heating weeks; GBR, Great Barrier Reef.
(DOCX)

S2 Table Parameters used for constructing survival-competency curves. All scale parameters were equal to 1. Mortality rate was constant and equal to 0.1 per day in all analyses shown in the text.
(DOCX)

S3 Table Results of a general linear model test that tested the effect of connectivity predictors on adult COTS densities observed in field surveys. Dispersion parameter for the fitted Tweedie distribution was 3.384. COTS, crown-of-thorns starfish.
(DOCX)

S4 Table Results of a general linear model that tested the effect of connectivity predictors on adult COTS densities obtained from CPUE during COTS eradication efforts. Model R^2^ = 20.82. COTS, crown-of-thorns starfish; CPUE, catch-per-unit effort.
(DOCX)

S1 Data Data used to generate the manuscript figures. (XLSX)

## Abbreviations

AMPTO: Association of Marine Park Tourism Operators
CoRTAD: Coral Reef Temperature Anomaly Database
COTS: crown-of-thorns starfish
CPUE: catch-per-unit effort
DHW: degree heating weeks
GBR: Great Barrier Reef
GBRMPA: Great Barrier Reef Marine Park Authority
RTNG: ReefTemp Next Generation

## References

[1] Carr MH, Neigel JE, Estes JA, Andelman S, Warner RR, Largier JL. Comparing marine and terrestrial ecosystems: implications for the design of coastal marine reserves. Ecol Appl. 2003;13(1):S90–S107.

[2] Hughes TP, Bellwood DR, Folke C, Steneck RS, Wilson J. New paradigms for supporting the resilience of marine ecosystems. Trends Ecol Evol. 2005;20(7):380–6. doi: 10.1016/j.tree.2005.03.022 16701400 10.1016/j.tree.2005.03.022

[3] Baker AC, Glynn PW, Riegl B. Climate change and coral reef bleaching: An ecological assessment of long-term impacts, recovery trends and future outlook. Estuar Coast Shelf Sci. 2008;80(4):435–71.

[4] Eakin CM, Morgan JA, Heron SF, Smith TB, Liu G, Alvarez-Filip L, et al Caribbean corals in crisis: record thermal stress, bleaching, and mortality in 2005. PLoS ONE. 2010;5(11):e13969 doi: 10.1371/journal.pone.0013969 21125021 10.1371/journal.pone.0013969 PMC2981599

[5] Hughes TP, Kerry JT, Álvarez-Noriega M, Álvarez-Romero JG, Anderson KD, Baird AH, et al Global warming and recurrent mass bleaching of corals. Nature. 2017;543(7645):373–7. doi: 10.1038/nature21707 28300113 10.1038/nature21707

[6] Nyström M, Folke C, Moberg F. Coral reef disturbance and resilience in a human-dominated environment. Trends Ecol Evol. 2000;15(10):413–7. 10998519 10.1016/s0169-5347(00)01948-0

[7] McClanahan TR, Donner SD, Maynard JA, MacNeil MA, Graham NA, Maina J, et al Prioritizing key resilience indicators to support coral reef management in a changing climate. PLoS ONE. 2012;7(8):e42884 doi: 10.1371/journal.pone.0042884 22952618 10.1371/journal.pone.0042884 PMC3430673

[8] Mumby PJ, Wolff NH, Bozec YM, Chollett I, Halloran P. Operationalizing the resilience of coral reefs in an era of climate change. Conserv Lett. 2014;7(3):176–87.

[9] Sale PF, Cowen RK, Danilowicz BS, Jones GP, Kritzer JP, Lindeman KC, et al Critical science gaps impede use of no-take fishery reserves. Trends Ecol Evol. 2005;20(2):74–80. doi: 10.1016/j.tree.2004.11.007 16701346 10.1016/j.tree.2004.11.007

[10] Cowen RK, Paris CB, Srinivasan A. Scaling of connectivity in marine populations. Science. 2006;311(5760):522–7. doi: 10.1126/science.1122039 16357224 10.1126/science.1122039

[11] Beger M, Grantham HS, Pressey RL, Wilson KA, Peterson EL, Dorfman D, et al Conservation planning for connectivity across marine, freshwater, and terrestrial realms. Biol Conserv. 2010;143(3):565–75.

[12] Mumby PJ, Elliott IA, Eakin CM, Skirving W, Paris CB, Edwards HJ, et al Reserve design for uncertain responses of coral reefs to climate change. Ecol Lett. 2011;14(2):132–40. doi: 10.1111/j.1461-0248.2010.01562.x 21105980 10.1111/j.1461-0248.2010.01562.x

[13] Man A, Law R, Polunin NV. Role of marine reserves in recruitment to reef fisheries: a metapopulation model. Biol Conserv. 1995;71(2):197–204.

[14] Nystrom M, Folke C. Spatial resilience of coral reefs. Ecosystems. 2001;4(5):406–17.

[15] Harrison HB, Williamson DH, Evans RD, Almany GR, Thorrold SR, Russ GR, et al Larval export from marine reserves and the recruitment benefit for fish and fisheries. Curr Biol. 2012;22(11):1023–8. doi: 10.1016/j.cub.2012.04.008 22633811 10.1016/j.cub.2012.04.008

[16] Maynard JA, Beeden R, Puotinen M, Johnson JE, Marshall P, Hooidonk R, et al Great Barrier Reef no-take areas include a range of disturbance regimes. Conserv Lett. 2015;9(3):191–9.

[17] Scheffer M, Carpenter SR, Lenton TM, Bascompte J, Brock W, Dakos V, et al Anticipating critical transitions. Science. 2012;338(6105):344–8. doi: 10.1126/science.1225244 23087241 10.1126/science.1225244

[18] Watson JR, Kendall BE, Siegel DA, Mitarai S. Changing seascapes, stochastic connectivity, and marine metapopulation dynamics. Am Nat. 2012;180(1):99–112. doi: 10.1086/665992 22673654 10.1086/665992

[19] Keppel G, Van Niel KP, Wardell-Johnson GW, Yates CJ, Byrne M, Mucina L, et al Refugia: identifying and understanding safe havens for biodiversity under climate change. Glob Ecol Biogeogr. 2012;21(4):393–404.

[20] Van Hooidonk R, Maynard J, Planes S. Temporary refugia for coral reefs in a warming world. Nat Clim Change. 2013;3(5):508–11.

[21] Hastings A, Cuddington K, Davies KF, Dugaw CJ, Elmendorf S, Freestone A, et al The spatial spread of invasions: new developments in theory and evidence. Ecol Lett. 2005;8(1):91–101.

[22] Treml EA, Roberts JJ, Chao Y, Halpin PN, Possingham HP, Riginos C. Reproductive output and duration of the pelagic larval stage determine seascape-wide connectivity of marine populations. Integr Comp Biol. 2012;52(4):525–37. doi: 10.1093/icb/ics101 22821585 10.1093/icb/ics101

[23] Skirving W, Heron M, Heron S. The hydrodynamics of a bleaching event: implications for management and monitoring In: Phinney JT, Hoegh-Guldberg O, Kleypas J, Skirving W, Strong A, editors. Coral Reefs and Climate Change: Science and Management. Washington, D. C: American Geophysical Union; 2006 p. 145–61.

[24] Chollett I, Mumby PJ, Cortés J. Upwelling areas do not guarantee refuge for coral reefs in a warming ocean. Mar Ecol-Prog Ser. 2010;416:47–56.

[25] Berkelmans R, De’ath G, Kininmonth S, Skirving WJ. A comparison of the 1998 and 2002 coral bleaching events on the Great Barrier Reef: spatial correlation, patterns, and predictions. Coral Reefs. 2004;23(1):74–83.

[26] Sweatman H, Delean S, Syms C. Assessing loss of coral cover on Australia's Great Barrier Reef over two decades, with implications for longer-term trends. Coral Reefs. 2011;30(2):521–31.

[27] Beeden R, Maynard J, Puotinen M, Marshall P, Dryden J, Goldberg J, et al Impacts and recovery from severe tropical Cyclone Yasi on the Great Barrier Reef. PLoS ONE. 2015;10(4):e0121272 doi: 10.1371/journal.pone.0121272 25874718 10.1371/journal.pone.0121272 PMC4398409

[28] Wolff NH, Wong A, Vitolo R, Stolberg K, Anthony KR, Mumby PJ. Temporal clustering of tropical cyclones on the Great Barrier Reef and its ecological importance. Coral Reefs. 2016;35(2):613–23.

[29] Fabricius KE, Okaji K, De'ath G. Three lines of evidence to link outbreaks of the crown-of-thorns seastar *Acanthaster planci* to the release of larval food limitation. Coral Reefs. 2010;29(3):593–605.

[30] Pratchett MS, Caballes CF, Rivera Posada JA, Sweatman HPA. Limits to understanding and managing outbreaks of crown-of-thorns starfish (*Acanthaster spp*.) In: Hughes RN, Hughes DJ, Smith IP, editors. Oceanogr Mar Biol. An Annual Review. 52: CRC Press; 2014 p. 133–200.

[31] De'ath G, Fabricius KE, Sweatman H, Puotinen M. The 27-year decline of coral cover on the Great Barrier Reef and its causes. Proc Natl Acad Sci U S A. 2012;109(44):17995–9. doi: 10.1073/pnas.1208909109 23027961 10.1073/pnas.1208909109 PMC3497744

[32] Vermeij M, Fogarty ND, Miller M. Pelagic conditions affect larval behavior, survival, and settlement patterns in the Caribbean coral *Montastraea faveolata*. Mar Ecol-Prog Ser. 2006;310(11):119–28.

[33] Treml EA, Halpin PN, Urban DL, Pratson LF. Modeling population connectivity by ocean currents, a graph-theoretic approach for marine conservation. Landsc Ecol. 2008;23(Suppl 1):19–36.

[34] Uthicke S, Logan M, Liddy M, Francis D, Hardy N, Lamare M. Climate change as an unexpected co-factor promoting coral eating seastar (*Acanthaster planci*) outbreaks. Sci Rep. 2015;5:8402 doi: 10.1038/srep08402 25672480 10.1038/srep08402 PMC4325318

[35] Mao Y, Luick JL. Circulation in the southern Great Barrier Reef studied through an integration of multiple remote sensing and in situ measurements. J Geophys Res Oceans. 2014;119(3):1621–43.

[36] Ortiz JC, Bozec Y-M, Wolff NH, Doropoulos C, Mumby PJ. Global disparity in the ecological benefits of reducing carbon emissions for coral reefs. Nat Clim Change. 2014;4:1090–4.

[37] Pratchett MS, Hoey AS, Wilson SK, Messmer V, Graham NA. Changes in biodiversity and functioning of reef fish assemblages following coral bleaching and coral loss. Diversity. 2011;3(3):424–52.

[38] Wooldridge SA. Water quality and coral bleaching thresholds: Formalising the linkage for the inshore reefs of the Great Barrier Reef, Australia. Mar Pollut Bull. 2009;58(5):745–51. doi: 10.1016/j.marpolbul.2008.12.013 19230930 10.1016/j.marpolbul.2008.12.013

[39] Devlin M, McKinna L, Alvarez-Romero J, Petus C, Abott B, Harkness P, et al Mapping the pollutants in surface riverine flood plume waters in the Great Barrier Reef, Australia. Mar Pollut Bull. 2012;65(4):224–35. 22469152 10.1016/j.marpolbul.2012.03.001

[40] Pratchett MS. Dynamics of an outbreak population of Acanthaster planci at Lizard Island, northern Great Barrier Reef (1995–1999). Coral Reefs. 2005;24(3):453–62.

[41] Hock K, Wolff NH, Condie SA, Anthony KRN, Mumby PJ. Connectivity networks reveal the risks of crown-of-thorns starfish outbreaks on the Great Barrier Reef. J Appl Ecol. 2014;51(5):1188–96.

[42] Moran PJ, De'Ath G. Estimates of the abundance of the crown-of-thorns starfish *Acanthaster planci* in outbreaking and non-outbreaking populations on reefs within the Great Barrier Reef. Mar Biol. 1992;113(3):509–15.

[43] McCook LJ, Ayling T, Cappo M, Choat JH, Evans RD, De Freitas DM, et al Adaptive management of the Great Barrier Reef: A globally significant demonstration of the benefits of networks of marine reserves. Proc Natl Acad Sci U S A. 2010;107(43):18278–85. doi: 10.1073/pnas.0909335107 20176947 10.1073/pnas.0909335107 PMC2972947

[44] Fabricius KE. Effects of terrestrial runoff on the ecology of corals and coral reefs: review and synthesis. Mar Pollut Bull. 2005;50(2):125–46. doi: 10.1016/j.marpolbul.2004.11.028 15737355 10.1016/j.marpolbul.2004.11.028

[45] Fabricius K, De’ath G, McCook L, Turak E, Williams DM. Changes in algal, coral and fish assemblages along water quality gradients on the inshore Great Barrier Reef. Mar Pollut Bull. 2005;51(1):384–98. 15757737 10.1016/j.marpolbul.2004.10.041

[46] De'ath G, Fabricius K. Water quality as a regional driver of coral biodiversity and macroalgae on the Great Barrier Reef. Ecol Appl. 2010;20(3):840–50. 20437968 10.1890/08-2023.1

[47] Brodie J, Kroon F, Schaffelke B, Wolanski E, Lewis S, Devlin M, et al Terrestrial pollutant runoff to the Great Barrier Reef: An update of issues, priorities and management responses. Mar Pollut Bull. 2012;65(4):81–100. 22257553 10.1016/j.marpolbul.2011.12.012

[48] Anthony K, Maynard JA, DIAZ-PULIDO G, Mumby PJ, Marshall PA, Cao L, et al Ocean acidification and warming will lower coral reef resilience. Glob Change Biol. 2011;17(5):1798–808.

[49] Mongin M, Baird ME, Tilbrook B, Matear RJ, Lenton A, Herzfeld M, et al The exposure of the Great Barrier Reef to ocean acidification. Nat Commun. 2016;7. 10.1038/ncomms10732 PMC4766391 26907171

[50] Steinberg C. Impacts of climate change on the physical oceanography of the Great Barrier Reef In: Johnson JE, Marshall PA, editors. Climate change and the Great Barrier Reef: a vulnerability assessment. Townsville, Australia: The Great Barrier Reef Marine Park Authority; 2007 p. 51–74.

[51] Munday P, Leis J, Lough J, Paris C, Kingsford M, Berumen M, et al Climate change and coral reef connectivity. Coral Reefs. 2009;28(2):379–95.

[52] Urban D, Keitt T. Landscape connectivity: a graph-theoretic perspective. Ecology. 2001;82(5):1205–18.

[53] Jordán F, Báldi A, Orci K-M, Racz I, Varga Z. Characterizing the importance of habitat patches and corridors in maintaining the landscape connectivity of a Pholidoptera transsylvanica (Orthoptera) metapopulation. Landsc Ecol. 2003;18(1):83–92.

[54] Bode M, Burrage K, Possingham HP. Using complex network metrics to predict the persistence of metapopulations with asymmetric connectivity patterns. Ecol Modell. 2008;214(2):201–9.

[55] Minor ES, Urban DL. A graph-theory frarmework for evaluating landscape connectivity and conservation planning. Conserv Biol. 2008;22(2):297–307. doi: 10.1111/j.1523-1739.2007.00871.x 18241238 10.1111/j.1523-1739.2007.00871.x

[56] Watson JR, Siegel DA, Kendall BE, Mitarai S, Rassweiller A, Gaines SD. Identifying critical regions in small-world marine metapopulations. Proc Natl Acad Sci U S A. 2011;108(43):E907–E13. doi: 10.1073/pnas.1111461108 21987813 10.1073/pnas.1111461108 PMC3203776

[57] Hock K, Mumby PJ. Quantifying the reliability of dispersal paths in connectivity networks. J Roy Soc Interface. 2015;12(105):20150013. 25716187 10.1098/rsif.2015.0013 PMC4387532

[58] With KA. The landscape ecology of invasive spread. Conserv Biol. 2002;16(5):1192–203.

[59] Chadès I, Martin TG, Nicol S, Burgman MA, Possingham HP, Buckley YM. General rules for managing and surveying networks of pests, diseases, and endangered species. Proc Natl Acad Sci U S A. 2011;108(20):8323–8. doi: 10.1073/pnas.1016846108 21536884 10.1073/pnas.1016846108 PMC3100963

[60] Minor ES, Gardner RH. Landscape connectivity and seed dispersal characteristics inform the best management strategy for exotic plants. Ecol Appl. 2011;21(3):739–49. 21639041 10.1890/10-0321.1

[61] Hock K, Wolff NH, Beeden R, Hoey J, Condie SA, Anthony KRN, et al Controlling range expansion in habitat networks by adaptively targeting source populations. Conserv Biol. 2016;30(4):856–66. doi: 10.1111/cobi.12665 26633077 10.1111/cobi.12665

[62] Heard GW, Thomas CD, Hodgson JA, Scroggie MP, Ramsey DS, Clemann N. Refugia and connectivity sustain amphibian metapopulations afflicted by disease. Ecol Lett. 2015;18(8):853–63. doi: 10.1111/ele.12463 26108261 10.1111/ele.12463

[63] Hughes TP, Rodrigues MJ, Bellwood DR, Ceccarelli D, Hoegh-Guldberg O, McCook L, et al Phase shifts, herbivory, and the resilience of coral reefs to climate change. Curr Biol. 2007;17(4):360–5. doi: 10.1016/j.cub.2006.12.049 17291763 10.1016/j.cub.2006.12.049

[64] Mumby PJ, Steneck RS, Adjeroud M, Arnold SN. High resilience masks underlying sensitivity to algal phase shifts of Pacific coral reefs. Oikos. 2015;125(5):644–55.

[65] GBRMPA. Great Barrier Reef Marine Park Zoning Plan 2003. Report ER. Townsville, QLD, Australia: Great Barrier Reef Marine Park Authority, 2004 ISBN9781876945237.

[66] van Nes EH, Scheffer M. Implications of spatial heterogeneity for catastrophic regime shifts in ecosystems. Ecology. 2005;86(7):1797–807.

[67] Foster NL, Paris CB, Kool JT, Baums IB, Stevens JR, Sanchez JA, et al Connectivity of Caribbean coral populations: complementary insights from empirical and modelled gene flow. Mol Ecol. 2012;21(5):1143–57. doi: 10.1111/j.1365-294X.2012.05455.x 22276913 10.1111/j.1365-294X.2012.05455.x

[68] Mumby PJ. Bleaching and hurricane disturbances to populations of coral recruits in Belize. Mar Ecol-Prog Ser. 1999;190:27–35.

[69] Mumby PJ, Van Woesik R. Consequences of ecological, evolutionary and biogeochemical uncertainty for coral reef responses to climatic stress. Curr Biol. 2014;24(10):R413–R23. doi: 10.1016/j.cub.2014.04.029 24845674 10.1016/j.cub.2014.04.029

[70] Palumbi SR, Barshis DJ, Traylor-Knowles N, Bay RA. Mechanisms of reef coral resistance to future climate change. Science. 2014;344(6186):895–8. doi: 10.1126/science.1251336 24762535 10.1126/science.1251336

[71] Mora C, Graham NA, Nyström M. Ecological limitations to the resilience of coral reefs. Coral Reefs. 2016;35(4):1271–80.

[72] Kwiatkowski L, Halloran PR, Mumby PJ, Stephenson DB. What spatial scales are believable for climate model projections of sea surface temperature? Clim Dyn. 2014;43(5–6):1483–96.

[73] Galindo HM, Olson DB, Palumbi SR. Seascape genetics: A coupled oceanographic-genetic model predicts population structure of Caribbean corals. Curr Biol. 2006;16(16):1622–6. doi: 10.1016/j.cub.2006.06.052 16920623 10.1016/j.cub.2006.06.052

[74] Liu G, Heron SF, Eakin CM, Muller-Karger FE, Vega-Rodriguez M, Guild LS, et al Reef-scale thermal stress monitoring of coral ecosystems: new 5-km global products from NOAA coral reef watch. Rem Sens. 2014;6(11):11579–606.

[75] Casey KS, Selig ER, Zhang D, Saha K, Krishnan A, McMichael E. The Coral Reef Temperature Anomaly Database (CoRTAD) Version 5—Global, 4 km Sea Surface Temperature and Related Thermal Stress Metrics for 1982–2012 (NCEI Accession 0126774). 2015.

[76] GBRMPA. Final report: 2016 coral bleaching event on the Great Barrier Reef. Townsville, QLD, Australia: Great Barrier Reef Marine Park Authority, 2017 0995373167.

[77] Ainsworth TD, Heron SF, Ortiz JC, Mumby PJ, Grech A, Ogawa D, et al Climate change disables coral bleaching protection on the Great Barrier Reef. Science. 2016;352(6283):338–42. doi: 10.1126/science.aac7125 27081069 10.1126/science.aac7125

[78] González-Rivero M, Beijbom O, Rodriguez-Ramirez A, Holtrop T, González-Marrero Y, Ganase A, et al Scaling up ecological measurements of coral reefs using semi-automated field image collection and analysis. Rem Sens. 2016;8(1):30.

[79] Caley M, Carr M, Hixon M, Hughes T, Jones G, Menge B. Recruitment and the local dynamics of open marine populations. Annu Rev Ecol Syst. 1996;27:477–500.

[80] Doropoulos C, Roff G, Bozec YM, Zupan M, Werminghausen J, Mumby PJ. Characterizing the ecological trade‐offs throughout the early ontogeny of coral recruitment. Ecol Monogr. 2016;86(1):20–44.

[81] Fox HE, Mous PJ, Pet JS, Muljadi AH, Caldwell RL. Experimental assessment of coral reef rehabilitation following blast fishing. Conserv Biol. 2005;19(1):98–107.

[82] Mora C. A clear human footprint in the coral reefs of the Caribbean. Proc R Soc B-Biol Sci. 2008;275(1636):767–73. 10.1098/rspb.2007.1472 PMC2596901 18182370

[83] Mora C, Aburto-Oropeza O, Bocos AA, Ayotte PM, Banks S, Bauman AG, et al Global human footprint on the linkage between biodiversity and ecosystem functioning in reef fishes. PLoS Biol. 2011;9(4):e1000606 doi: 10.1371/journal.pbio.1000606 21483714 10.1371/journal.pbio.1000606 PMC3071368

[84] Condie S, Hepburn M, Mansbridge J, editors. Modelling and visualisation of connectivity on the Great Barrier Reef. Proceedings of the 12th International Coral Reef Symposium; 2012 9–13 July 2012; Cairns, Australia.

[85] White JW, Morgan SG, Fisher JL. Planktonic larval mortality rates are lower than widely expected. Ecology. 2014;95(12):3344–53.

[86] Sammarco PW, Andrews JC. The Helix experiment: differential localized dispersal and recruitment patterns in Great Barrier Reef corals. Limnol Oceanogr. 1989;34(5):896–912.

[87] Andutta FP, Kingsford MJ, Wolanski E. ‘Sticky water’enables the retention of larvae in a reef mosaic. Estuar Coast Shelf Sci. 2012;101:54–63.

[88] Wolanski E, Kingsford MJ. Oceanographic and behavioural assumptions in models of the fate of coral and coral reef fish larvae. J Roy Soc Interface. 2014;11(98):20140209. 24966233 10.1098/rsif.2014.0209 PMC4233683

[89] Garde L, Spillman C, Heron S, Beeden R. Reef Temp Next Generation: A new operational system for monitoring reef thermal stress. J Oper Oceanogr. 2014;7(1):21–33.

[90] Harrison HB, Pratchett MS, Messmer V, Saenz-Agudelo P, Berumen ML. Microsatellites reveal genetic homogeneity among outbreak populations of crown-of-thorns starfish (*Acanthaster cf*. *solaris*) on Australia’s Great Barrier Reef. Diversity. 2017;9(1):16.

[91] Powers DM. Evaluation: from precision, recall and F-measure to ROC, informedness, markedness and correlation. J Mach Learn Tech. 2011;2(1):37–63.

[92] Shono H. Application of the Tweedie distribution to zero-catch data in CPUE analysis. Fish Res. 2008;93(1):154–62.

