# Supplementary figures and images for: Connectivity and systemic resilience of the Great Barrier Reef
Source: PLoS Biol. 2017 Nov 28;15(11):e2003355. doi: 10.1371/journal.pbio.2003355 (PMC5705071; doi:10.1371/journal.pbio.2003355)

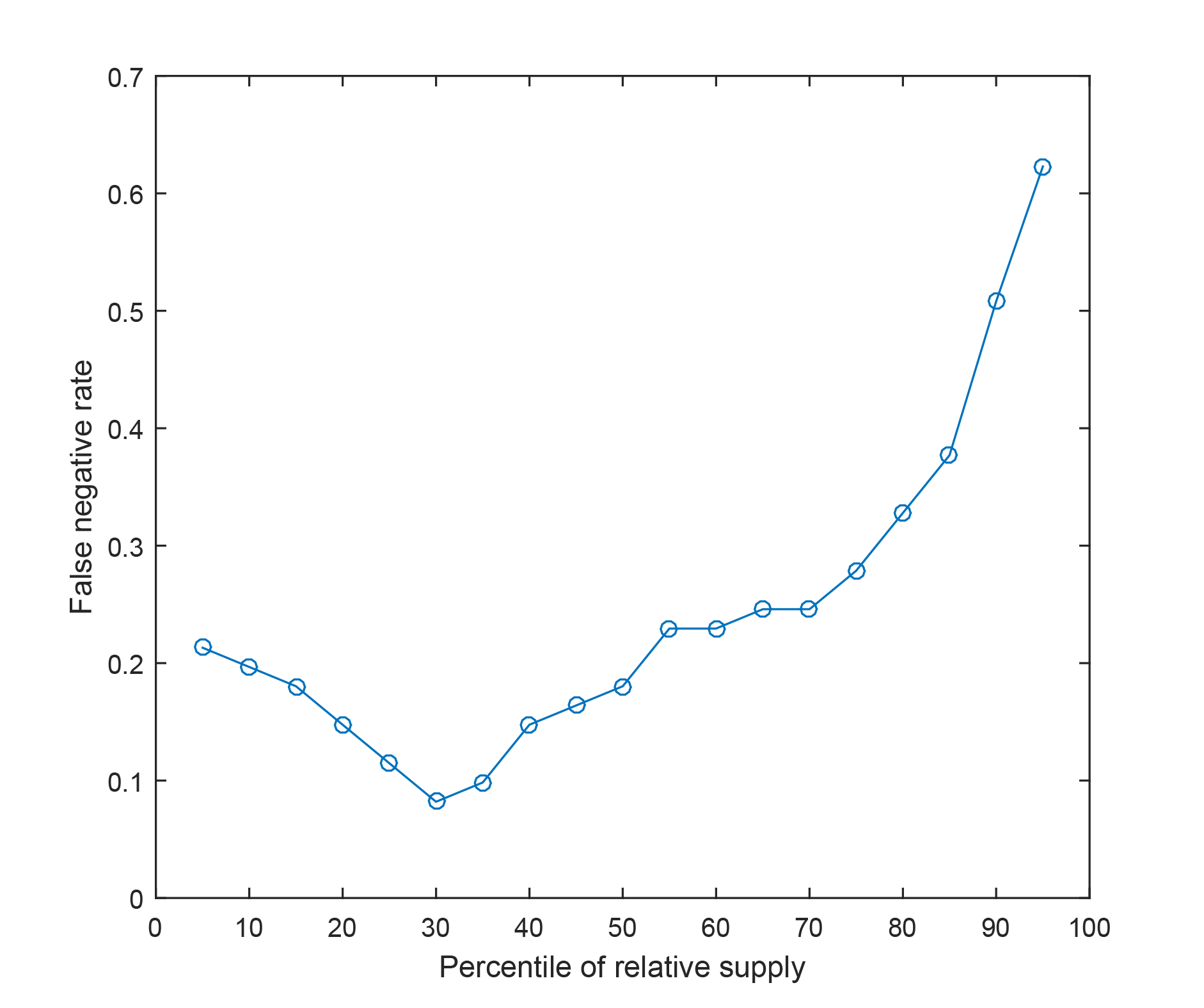

Supplement: S1 Fig — The threshold of 30th percentile was found to perform the best as it gives the lowest rate of false negatives (reefs that are classified as low risk due to low supply of larvae but also had adult COTS outbreaks in the surveys). This percentile was then used as a threshold to classify reefs that will have low risk of COTS outbreaks. COTS, crown-of-thorns starfish. (TIF) [file pbio.2003355.s001.tif]

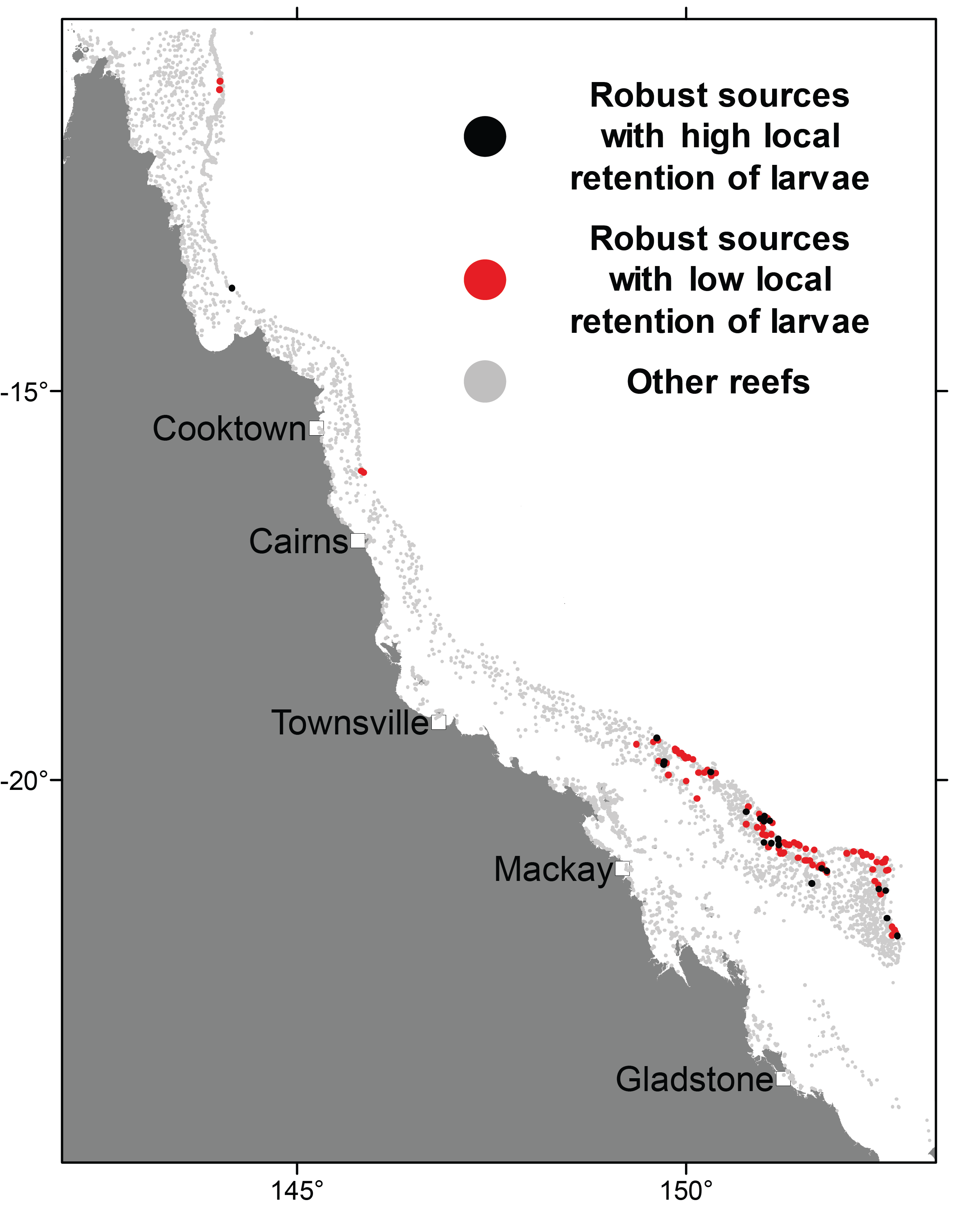

Supplement: S2 Fig — Black circles represent robust sources that have had consistently high local retention of larvae in dispersal simulations when compared to the GBR-wide average; red circles, well represented in the outer shelf regions, represent robust sources that have had below average local retention levels. The majority (80%) of the robust sources have low levels of local retention, possibly due to high flushing regimes. GBR, Great Barrier Reef. (TIF) [file pbio.2003355.s002.tif]

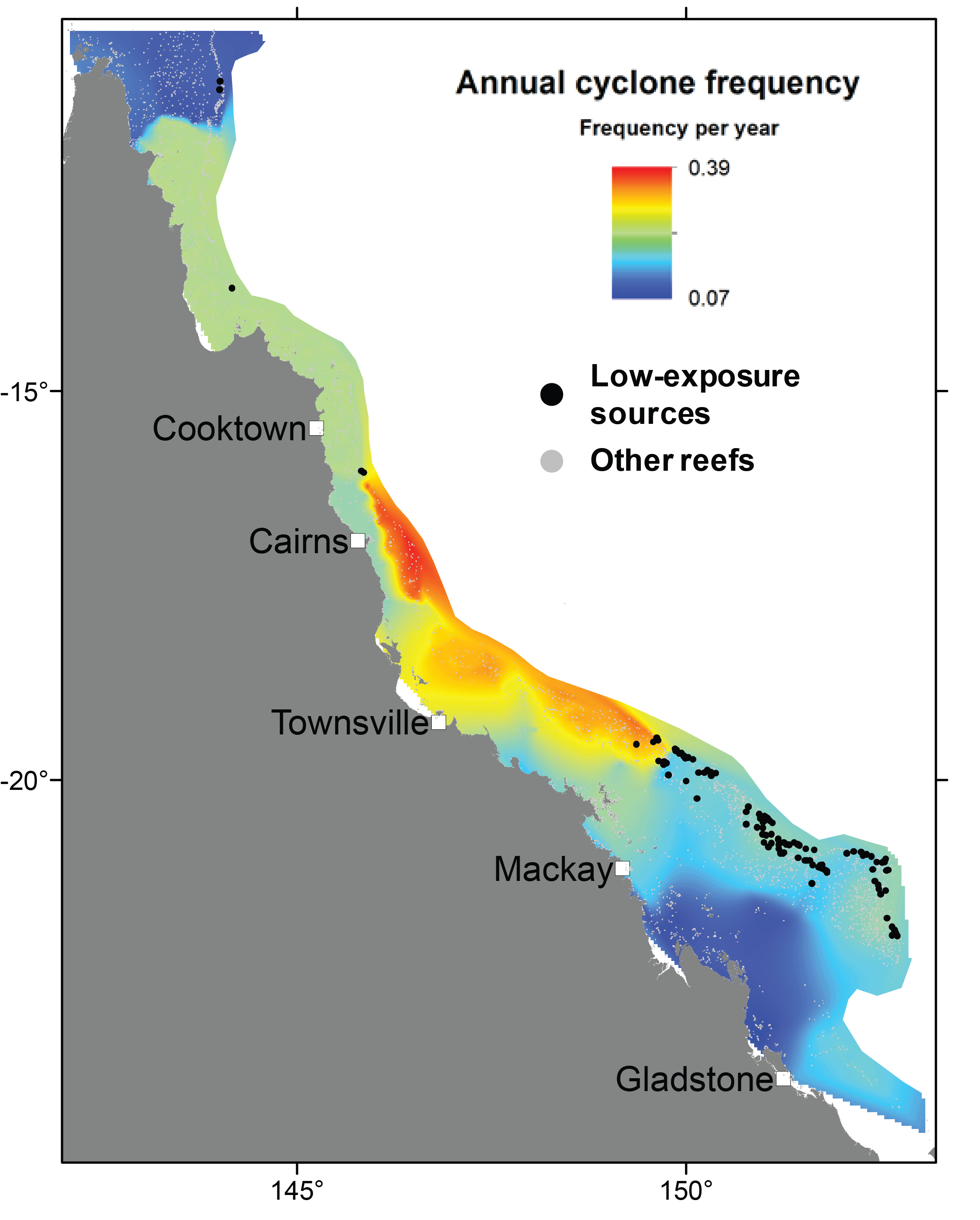

Supplement: S3 Fig — Robust sources tend to be located outside of the regions with high expected cyclone frequency (coloured background; adapted from data presented in Wolff et al. [28]). GBR, Great Barrier Reef. (TIF) [file pbio.2003355.s003.tif]

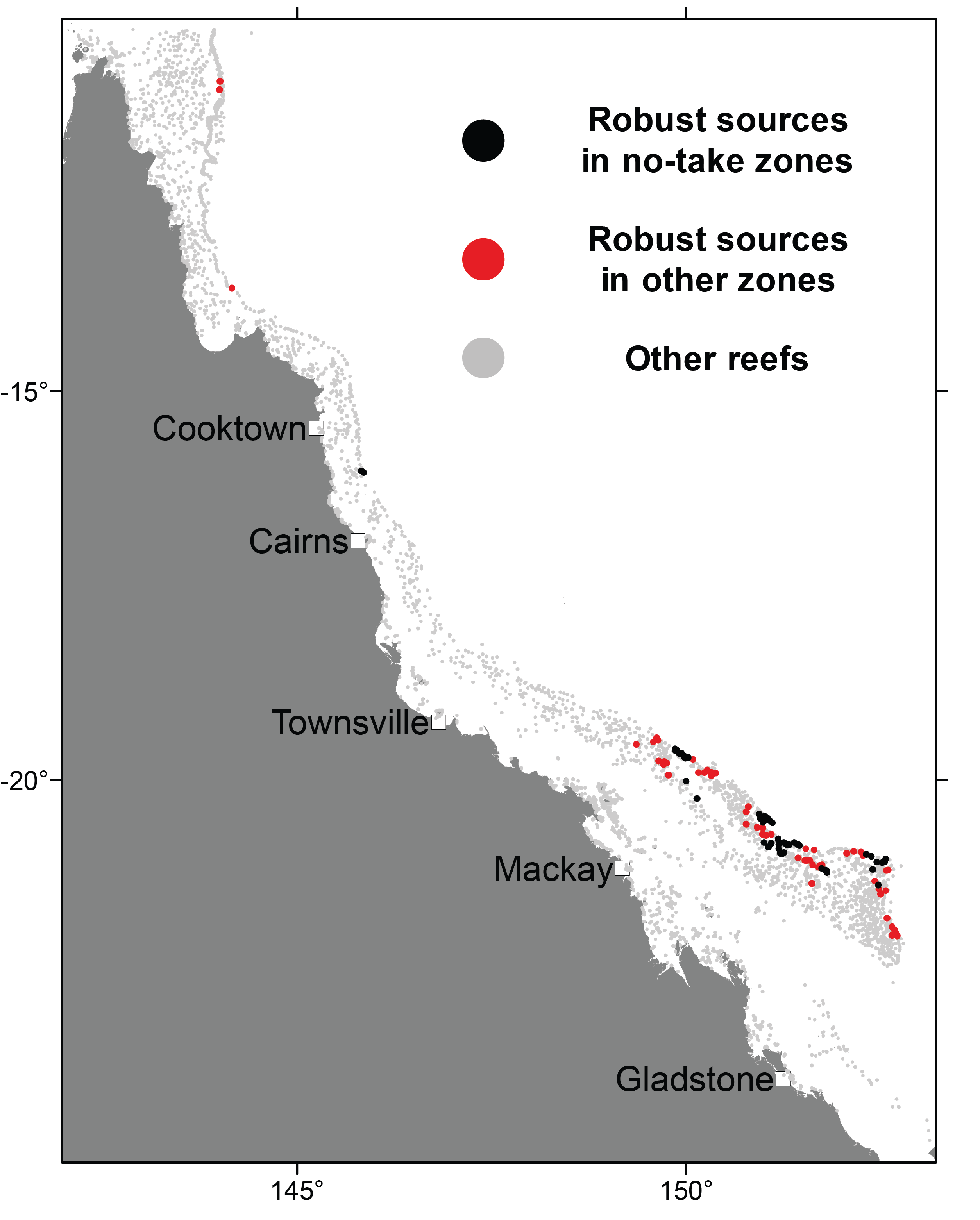

Supplement: S4 Fig — Black circles represent robust sources located in no-take zones; red circles represent robust sources that are not located in no-take zones. Nearly half (46%) of the robust sources are already located in no-take zones and awarded the highest level of protection under the current GBR zoning plan [65]. GBR, Great Barrier Reef. (TIF) [file pbio.2003355.s004.tif]
