# Supplementary material for: Connectivity and systemic resilience of the Great Barrier Reef
Source: PLoS Biol. 2017 Nov 28;15(11):e2003355. doi: 10.1371/journal.pbio.2003355 (PMC5705071; doi:10.1371/journal.pbio.2003355)
Supplement: S1 Table — COTS, crown-of-thorns starfish; DHW, degree heating weeks; GBR, Great Barrier Reef. (DOCX) [file pbio.2003355.s005.docx]

**S1 Table. Key assumptions and rationales behind the 3 criteria used to identify robust sources.** COTS, crown-of-thorns starfish; DHW, degree heating weeks; GBR, Great Barrier Reef.

| **Criterion** | **Assumption** | **Rationale** |
| --- | --- | --- |
| 1) Reefs need to be strong, consistent sources of larvae | That larval supply among reefs is an ecologically-relevant process for the recolonization of corals after large disturbance events. | a) Larval supply is likely to have the greatest demographic impact when adult population size is low and space is not limiting (i.e., post-settlement density dependence is weakest) [[1](#_ENREF_1)].  b) While genetic measures of gene flow do not reflect specific demographic rates they can identify areas where larval flux is blocked, such as if coral replenishment was solely owing to self-recruitment. Yet, repeated genetic studies of broadcast spawning corals, including those on the GBR, find high levels of gene flow across the system [[2](#_ENREF_2), [3](#_ENREF_3)].  c) Even corals that brood their larvae, which tend to have very limited pelagic durations, exhibit connectivity over scales of 10s to 100s of km within a few generations [[4](#_ENREF_4)].  d) Other high resolution models of larval dispersal on the GBR find that the self-seeding rate of corals is less than 2% [[5](#_ENREF_5)].  e) We note that the importance of larval supply will vary across the GBR and will likely be less important in some degraded inshore environments where post-settlement mortality is likely to be extremely high. Patterns of post-settlement mortality have not yet been mapped. |
| 2) Low risk of thermal stress / coral bleaching | Reefs will not have experienced DHW >6 | a) While some reefs will experience bleaching at around 4 DHW, coral mortality is virtually absent at this stress level [[6](#_ENREF_6)]. Observations from recent bleaching events indicate that coral morality can be expected after a thermal stress of about 6 DHW, but is otherwise low [[7](#_ENREF_7)].  b) It is true that corals experiencing chronically warmer conditions are better acclimated (and perhaps adapted) to severe bleaching conditions when they occur [[8-10](#_ENREF_8)]. Thus, corals used to cooler conditions tend to fare poorly when subjected to severe warming. However, the key element of our criterion is that the selected reefs rarely (if ever) experience such thermal stress. One of the few studies of coral mortality post-bleaching found that while acclimation temperature is a predictor of mortality, the dominant predictor is the magnitude of the stress event (DHW) [[11](#_ENREF_11)]. |
| 3) Low risk of being infected by and transmitting COTS | Model predictions of COTS larvae can be divided into ‘low’ and ‘high’ categories around the mean, yet translate into ecologically important impacts | a) It is widely reported that outbreaks of COTS move as a ‘wave’ down the GBR [[12](#_ENREF_12)]. Such patterns have been shown to be predictable using connectivity models [[13](#_ENREF_13)].  b) The process of COTS’ migration from reef to reef involves new colonisation events, it is likely that larval supply is a key demographic driver [[12](#_ENREF_12), [14](#_ENREF_14), [15](#_ENREF_15)]. Any post-settlement density dependence, such as competition, is likely to be low at this stage of population build up.  c) Comparison of our model predictions with two independent field data sets reveals that COTS densities were more than four times lower and outbreaks nearly 14 times less likely on reefs categorised as having a low risk of importing COTS larvae. |

**References:**

1. Caley M, Carr M, Hixon M, Hughes T, Jones G, Menge B. Recruitment and the local dynamics of open marine populations. Annu Rev Ecol Syst. 1996;27:477-500.

2. Foster NL, Paris CB, Kool JT, Baums IB, Stevens JR, Sanchez JA, et al. Connectivity of Caribbean coral populations: complementary insights from empirical and modelled gene flow. Mol Ecol. 2012;21(5):1143-57.

3. Lukoschek V, Riginos C, van Oppen MJ. Congruent patterns of connectivity can inform management for broadcast spawning corals on the Great Barrier Reef. Mol Ecol. 2016;25(13):3065-80.

4. van Oppen MJ, Lutz A, De'ath G, Peplow L, Kininmonth S. Genetic traces of recent long-distance dispersal in a predominantly self-recruiting coral. PLoS ONE. 2008;3(10):e3401.

5. Wolanski E, Kingsford MJ. Oceanographic and behavioural assumptions in models of the fate of coral and coral reef fish larvae. J Roy Soc Interface. 2014;11(98):20140209.

6. Eakin CM, Morgan JA, Heron SF, Smith TB, Liu G, Alvarez-Filip L, et al. Caribbean corals in crisis: record thermal stress, bleaching, and mortality in 2005. PLoS ONE. 2010;5(11):e13969.

7. GBRMPA. Final report: 2016 coral bleaching event on the Great Barrier Reef. Townsville, QLD, Australia: Great Barrier Reef Marine Park Authority, 2017 0995373167.

8. Ainsworth TD, Heron SF, Ortiz JC, Mumby PJ, Grech A, Ogawa D, et al. Climate change disables coral bleaching protection on the Great Barrier Reef. Science. 2016;352(6283):338-42.

9. Ulstrup KE, Berkelmans R, Ralph PJ, van Oppen MJH. Variation in bleaching sensitivity of two coral species across a latitudinal gradient on the Great Barrier Reef: the role of zooxanthellae. Mar Ecol-Prog Ser. 2006;314:135-48.

10. Seneca FO, Palumbi SR. The role of transcriptome resilience in resistance of corals to bleaching. Mol Ecol. 2015;24(7):1467-84.

11. Mumby PJ, Elliott IA, Eakin CM, Skirving W, Paris CB, Edwards HJ, et al. Reserve design for uncertain responses of coral reefs to climate change. Ecol Lett. 2011;14(2):132-40.

12. Pratchett MS, Caballes CF, Rivera Posada JA, Sweatman HPA. Limits to understanding and managing outbreaks of crown-of-thorns starfish (*Acanthaster spp.*). In: Hughes RN, Hughes DJ, Smith IP, editors. Oceanogr Mar Biol. An Annual Review. 52: CRC Press; 2014. p. 133-200.

13. Hock K, Wolff NH, Condie SA, Anthony KRN, Mumby PJ. Connectivity networks reveal the risks of crown-of-thorns starfish outbreaks on the Great Barrier Reef. J Appl Ecol. 2014;51(5):1188-96.

14. Benzie J, Stoddart J. Genetic structure of outbreaking and non-outbreaking crown-of-thorns starfish (*Acanthaster planci*) populations on the Great Barrier Reef. Mar Biol. 1992;112(1):119-30.

15. Harrison HB, Pratchett MS, Messmer V, Saenz-Agudelo P, Berumen ML. Microsatellites reveal genetic homogeneity among outbreak populations of crown-of-thorns starfish (*Acanthaster cf. solaris*) on Australia’s Great Barrier Reef. Diversity. 2017;9(1):16.
