# Supplementary material for: Connectivity and systemic resilience of the Great Barrier Reef
Source: PLoS Biol. 2017 Nov 28;15(11):e2003355. doi: 10.1371/journal.pbio.2003355 (PMC5705071; doi:10.1371/journal.pbio.2003355)
Supplement: S2 Table — All scale parameters were equal to 1. Mortality rate was constant and equal to 0.1 per day in all analyses shown in the text. (DOCX) [file pbio.2003355.s006.docx]

**S2 Table.** **Parameters used for constructing survival-competency curves.** All scale parameters were equal to 1. Mortality rate was constant and equal to 0.1 per day in all analyses shown in the text.

| **Days until 50% of larvae competent (shape parameters *k* of gamma functions)** | **Maximum survival of larvae (days)** |
| --- | --- |
| 0.5 | 1 |
| 1 | 2 |
| 1.5 | 3 |
| 2.5 | 5 |
| 5 | 10 |
| 7.5 | 15 |
| 10 | 20 |
| 12.5 | 25 |
| 15 | 30 |
| 17.5 | 30 |
| 20 | 30 |
| 22.5 | 30 |
| 25 | 30 |
