# Supplementary material for: Connectivity and systemic resilience of the Great Barrier Reef
Source: PLoS Biol. 2017 Nov 28;15(11):e2003355. doi: 10.1371/journal.pbio.2003355 (PMC5705071; doi:10.1371/journal.pbio.2003355)
Supplement: S3 Table — Dispersion parameter for the fitted Tweedie distribution was 3.384. COTS, crown-of-thorns starfish. (DOCX) [file pbio.2003355.s007.docx]

**S3 Table.** **Results of a general linear model test that tested the effect of connectivity predictors on adult COTS densities observed in field surveys.** Dispersion parameter for the fitted Tweedie distribution was 3.384. COTS, crown-of-thorns starfish.

| **Parameter** | **Estimate** | **Std. Error** | **t** | ***p*(>\|t\|)** |
| --- | --- | --- | --- | --- |
| Intercept | 14.04 | 130.8 | 0.107 | 0.915 |
| Longitude | -0.020 | 0.941 | -0.021 | 0.983 |
| Latitude | 0.822 | 0.455 | 1.806 | 0.073 |
| Date of survey | 0.001 | 0.001 | 0.431 | 0.668 |
| Coral cover | -2.065 | 2.001 | -1.032 | 0.304 |
| Reef size | 0.005 | 0.004 | 1.174 | 0.243 |
| COTS external larval supply | 2.342 | 0.507 | 4.622 | 9.08e-6 *** |
| COTS local larval retention | 0.002 | 0.442 | 0.004 | 0.997 |
