# Supplementary material for: Connectivity and systemic resilience of the Great Barrier Reef
Source: PLoS Biol. 2017 Nov 28;15(11):e2003355. doi: 10.1371/journal.pbio.2003355 (PMC5705071; doi:10.1371/journal.pbio.2003355)
Supplement: S4 Table — Model R2 = 20.82. COTS, crown-of-thorns starfish; CPUE, catch-per-unit effort. (DOCX) [file pbio.2003355.s008.docx]

**S4 Table.** **Results of a general linear model that tested the effect of connectivity predictors on adult COTS densities obtained from CPUE during COTS eradication efforts**. Model R^2^ = 20.82. COTS, crown-of-thorns starfish; CPUE, catch-per-unit effort.

| **Parameter** | **DF** | **Adj SS** | **Adj MS** | **F** | ***p*** |
| --- | --- | --- | --- | --- | --- |
| Longitude | 1 | 0.181 | 0.181 | 1.04 | 0.310 |
| Latitude | 1 | 0.012 | 0.012 | 0.07 | 0.790 |
| Date of cull | 1 | 0.512 | 0.512 | 2.95 | 0.089 |
| Coral cover | 1 | 0.013 | 0.013 | 0.07 | 0.787 |
| Reef size | 1 | 0.051 | 0.051 | 0.30 | 0.588 |
| Boat ID | 1 | 0.009 | 0.009 | 0.05 | 0.823 |
| COTS external larval supply | 1 | 1.198 | 1.198 | 6.92 | 0.010 ** |
| COTS local larval retention | 1 | 0.252 | 0.252 | 1.45 | 0.231 |
| Error | 85 | 14.726 | 0.173 |  |  |
| Total | 93 | 18.599 |  |  |  |
